# Comparative Study of the Chemical Constituents and Bioactivities of the Extracts from Fruits, Leaves and Root Barks of *Lycium barbarum*

**DOI:** 10.3390/molecules24081585

**Published:** 2019-04-22

**Authors:** Xiao Xiao, Wei Ren, Nan Zhang, Tao Bing, Xiangjun Liu, Zhenwen Zhao, Dihua Shangguan

**Affiliations:** 1 Beijing National Laboratory for Molecular Sciences, Key Laboratory of Analytical Chemistry for Living Biosystems, CAS Research/Education Center for Excellence in Molecular Sciences, Institute of Chemistry, Chinese Academy of Sciences, Beijing 100190, China; xiaoxiao@iccas.ac.cn (X.X.); renwei1991@iccas.ac.cn (W.R.); hszhang@iccas.ac.cn (N.Z.); bingtao@iccas.ac.cn (T.B.); xjliu@iccas.ac.cn (X.L.); 2 University of the Chinese Academy of Sciences, Beijing 100049, China

**Keywords:** chemical compounds, antioxidative activities, organs of *Lycium barbarum*, UPLC-MS, comparative study

## Abstract

The fruits, leaves and root barks of *L. barbarum* plant are widely used as functional foods and as ingredients in traditional Chinese prescriptions and patent medicines. They are considered to have different pharmacological activities and health benefits because of their diverse constituents. Here, the chemical constituents of the extracts from fruits, leaves and root barks of *L. barbarum* were compared by ultra-high performance liquid chromatography coupled with high resolution mass spectrometry (UPLC-HR-MS). A total of 131 compounds were identified and seven of them were quantified. Among them, 98, 28 and 35 constituents were detected in fruits, leaves and root barks respectively. Dicaffeoylspermidine/spermine derivatives were the most detected compounds (74/131); among them, dicaffeoylspermine isomers and propionyl-dicaffeoylspermidine were found in root barks in very large amounts (e.g., kukoamine B = 10.90 mg/g dry powder); dicaffeoyl-spermidine isomers were detected in fruits/leaves in a high amount, and many of their glycosylated derivatives were mainly detected in fruits. In addition, six saponins from *L. barbarum* fruits were reported for the first time, and 5,6-dihydrosolasonine was reported for the first time in plants. The activity assays showed that the root bark extract possessed the strongest antioxidative activity and cytotoxicity, which was presumed due to the large amount of dicaffeoylspermine/spermidines in root barks. Fourteen potential bioactive components from fruits were identified by a target cell-based screening method. These results will help to understand the different biological activities of these three parts of *L. barbarum* plant and will benefit the discovery of new functional components.

## 1. Introduction

*Lycium barbarum* L. (*L. barbarum*), known as “goji” or Chinese wolfberry, belongs to the family of *Solanaceae* and is widely cultivated in China. Three parts of *L. barbarum* plant including fruits, leaves and root barks, have been used as functional foods and traditional Chinese medicinal herbs in China for centuries [1,2,3] and nowadays are being widely consumed all over the world. The fruits (goji berries, Chinese name: gouqizi) are reported to have multiple effects, such as anti-aging, neuroprotection, anti-fatigue, hypoglycemic, antiproliferative activity and cytoprotection, immunomodulation and antioxidant properties [3,4,5] and are being most widely used in foods and traditional medicines. The leaves, called “tianjingcao” in traditional Chinese medicine, have the benefits of alleviating mineral deficiency, combating heat distress, quenching thirst, dispelling wind, and enhancing eyesight, and have been widely used as tea, vegetables and medicines [6]. The root barks (Lycii Cortex Radicis, Chinese name: digupi or jikoppi) are officially listed in the Chinese Pharmacopoeia for the treatment of diabetes mellitus, night sweats, coughs, hematemesis, hypertension, and ulcers [7,8]. These three parts are considered to have different pharmacological activities and health benefits, and are widely used in different prescriptions and traditional Chinese patent medicines. The different biological activities of the three parts of *L. barbarum* plant are attributed to their different functional components. Many studies have identified and even quantified various chemical components in the three parts of *Lycium genus* plants individually, especially in fruits, including polysaccharides, peptide, alkaloids, flavonoids, terpenes, organic acids, lignans, phenolic amides, carotenoids, etc [3,9,10,11]. For example, Patsilinakos et al. have studied the carotenoid content in goji berries cultivated in Italy, evaluating the differences among varieties, harvesting periods, seasons, and extracting procedures by colorimetric and high performance liquid chromatograph-diode array detector (HPLC-DAD) analyses [12]. Inbaraj et al. have identified a total of 52 phenolic acids and flavonoids in *Lycium barbarum* Linnaeus by HPLC-DAD coupled electrospray ionization mass spectrometry (HPLC-DAD-ESI-MS) [13]. Mocan et al. have quantified eight phenolic acids and eleven flavonoids in *L. barbarum* and *Lycium chinense* Mill leaves [14]. However, to the best of our knowledge, few study has compared the difference of the chemical components of these three parts. The comparative study of the chemical components will help to understand the unique biological activities of these three parts and demonstrate their potential functional components.

Because of the excellent physical separation capability of UPLC, and the powerful identification ability of high-resolution mass spectrometry (HR-MS), UPLC coupled HR-MS (UPLC-HR-MS) has been extensively used in systematically identifying and quantifying components in complex samples [15]. For example, Patras et al. used UPLC-HR-MS to profile and quantify the regioisomeric caffeoyl glucoses in goji berry fruits [16]. Mocan et al. have employed UPLC coupled quadrupole-time of flight mass spectrometer (UPLC-QTOF-MS) to study the bioactive constituents of two Romanian Goji (*L. barbarum*) berries cultivars and evaluated their antioxidant and enzyme inhibitory properties [17]. Recently, UPLC coupled hybrid triple quadrupole linear ion trap mass spectrometer (UPLC-Qtrap-MS) with targeted multiple reactions monitoring (MRM) mode shows high sensitivity, specificity, and selectivity in the simultaneous identification and quantitation of compounds in large concentration ranges and complex matrices [18]. 

The aim of this study is to compare the chemical constituents and the bioactivities of the ethanol/water (70/30, *v*/*v*) extracts from the fruits, leaves and root barks of *L. barbarum*. A total of 131 compounds were identified by UPLC coupled Orbitrap mass spectrometry (UPLC-Orbitrap-MS). Five compounds in fruits, one compound in root barks and three compounds in leaves were quantified by UPLC-Qtrap-MS. The antioxidative activity of these extracts was evaluated in buffer and in cells, respectively, and the cytotoxicity of the extracts was tested. In addition, a compound database was constructed based on the chemical constituents of fruits extract, based on which, the potential bioactive components in fruits of *L. barbarum* were identified by the target cell-based screening method.

## 2. Results and Discussion

### 2.1. Isolation and Identification of 5,6-dihydrosolasonine from Fruits

**Figure 1:**
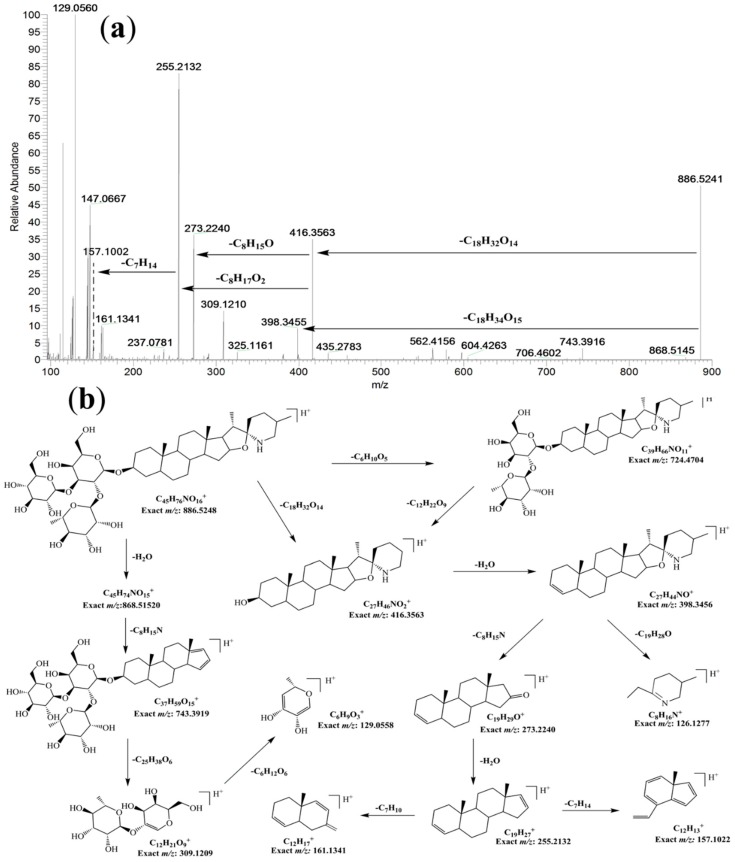
The HR-Orbitrap MS/MS spectrum (**a**) and proposed fragmentation pathway (**b**) of 5,6-dihydrosolasonine.

In our preliminary experiment of the qualitative analysis of fruits extract by UPLC-HR-MS, two compounds displaying [M + H]^+^ ions at *m/z* 884.5084 and 886.5241 attracted our attention because they had not been previously reported in *L. barbarum*. The compound with [M + H]^+^ ion at *m/z* 884.5084 was assigned to the molecular formula of C_45_H_74_O_16_N, with a mass error of 1.13 ppm. Based on its fragmentation pattern, this compound was identified to be the spirosolane-type glycoalkaloid, solasonine [19], which was further confirmed by comparing its retention time in UPLC (Figure S1) and MS/MS fragmentation ions with a solasonine standard (Figure S2). The fragment ions at *m/z* 722.4544 [M + H − C_6_H_10_O_5_]^+^, 576.3957 [M + H − C_6_H_10_O_5_ − C_6_H_10_O_4_]^+^, 414.3403 [M + H − C_6_H_10_O_5_ − C_6_H_10_O_4_ − C_6_H_10_O_5_]^+^, correspond to the successive losses of glucosyl, rhamnosyl and glucosyl. The production at *m/z* 414.3403 was ascribed to the aglycone ion of the steroidal glycoalkaloid. The fragment ion at *m/z* 271.2054 originates from the neutral loss (143 Da) of the E-ring and nitrogen-containing F-ring moiety from the ion at *m/z* 414.3403. The ion at *m/z* 253.1950 was formed by the neutral loss of H_2_O from the ion at *m/z* 271.2054 (Figure S2). The compound with [M + H]^+^ ion at *m/z* 886.5152 was assigned a molecular formula of C_45_H_76_O_16_N with a mass error of 0.75 ppm, which has two more hydrogens than solasonine. Furthermore, most of its fragmentation ions showed two Da higher (Figure 1, 868.5034; 722.4475; 578.4109; 416.3563; 398.3408; 273.2205; 255.2102) than those of solasonine (Figure S2, 866.4896; 720.4325; 576.3898; 414.3403; 396.3259; 271.2054; 253.1949), suggesting the loss of the same neutral fragments during the MS fragmentation (e.g., the sugar moiety). 

This result suggests that the new compound has a similar structure to solasonine except for two more hydrogens on the aglycone. Based on the fragmentation ions of both compounds and the reported fragmentation pathway of solasonine [19], the new compound could be 5,6-dihydrosolasonine, which differs from solasonine by the C-C single bond at position 5,6 in the B-ring of the steroidal skeleton. The proposed aglycone of 5,6-dihydrosolasonine (named as soladulcidine) has been reported in other two glycoalkaloids (soladulcine A/B) isolated from *Solanum dulcamara*, which consist of chacotriose/lycotetraose and soladulcidine joined through a β-glycosidic bond [20]. The proposed fragmentation pathway for 5,6-dihydrosolasonine is shown in Figure 1. Furthermore, 5,6-dihydrosolasonine (20 mg, white powder, UV λ_max_: 225 nm) was isolated from dried fruit of *L. barbarum* (5 kg) and characterized by MS and ^13^C-NMR. The NMR data (Table S1) showed that the peaks at 140.6 ppm and 121.4 ppm in the ^13^C-NMR spectrum of solasonine disappeared in the ^13^C-NMR spectrum of 5,6-dihydrosolasonine, while two new peaks appeared at 43.06 ppm and 28.77 ppm, which correspond to the change of the double bound to a C-C single bond at position 5,6 in the B-ring of the steroidal skeleton. In addition, soladulcidine (22*R*, 25*R*) has a stereoisomer, tomatidine (22*S*, 25*S*), the aglycone of α-tomatine found in the stems and leaves of tomato plants. The ^13^C-NMR peak at 33.73 ppm (C23) and 45.87 ppm (C26) further confirmed that the aglycone of 5,6-dihydrosolasonine is soladulcidine [21].

Glycoalkaloids are nitrogen-containing steroidal glycosides, generally found in plants of the Solanaceae, such as tomato, potato, and aubergine [22]. Solasonine and solamargine are two major steroidal glycoalkaloids, which have been found in 200 Solanum species [23,24,25]. They are water soluble triglycosides with the same aglycone (solasodine) and different trioses (solatriose and chacotriose) [25]. Solasodine is one of the main aglycone of glycoalkaloids, and has been used as raw material for steroidal drugs. Although *L. barbarum* belongs to the Solanaceae family, solasonine has not been previously reported in *L. barbarum*. A ring E-opened dihydro-derivative of solasonine has been reported by Weissenberg et al. [26,27] which has the same molecular weight as 5,6-dihydros- olasonine, but should have different fragmentation ions from those shown in Figure 1. As far as we know, 5,6-dihydrosolasonine has not been previously identified in any plant. The isolated 5,6-dihydrosolasonine was used as a standard substance for the following quantitative study.

### 2.2. Multi-Component Analysis of Extracts by UPLC-HR-MS

**Figure 2:**
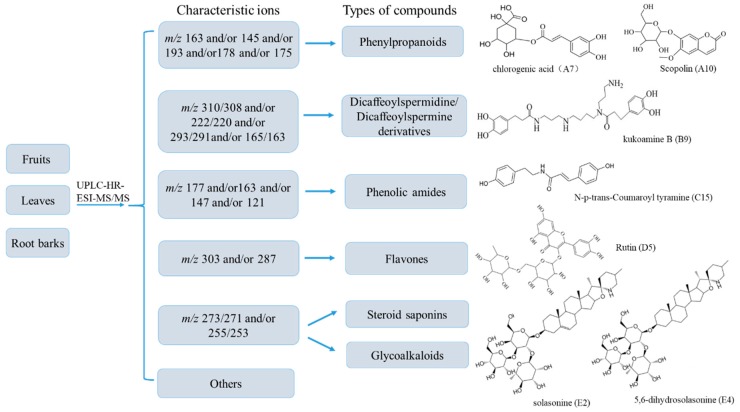
A diagram for rapid classification and tentative identification of chemical constituents in the extracts of fruits, leaves and root barks of *L. barbarum* by UPLC-HR-MS, and the structures of standard compounds in different classes.

Under the optimized UPLC-HR-MS experimental conditions, the accurate mass and composition for the precursor ions and product ions from the extracts of fruits, leaves and root barks were analyzed respectively using Xcalibur^TM^ 3.0 (Thermo Fisher) software in both positive and negative ionization modes. Internal calibration by infusion of a calibrant achieved a typical mass accuracy within 10 ppm. The identification of the compounds in extracts was performed based on the retention time, high resolution MS/MS data, isotope abundance, fragment product ions, literature data, databases (Reaxys, PubMed, Mass Bank, Chemspider, etc.) and standard substances. The fragmentation patterns (Figure 1 and Figures S2–S6) of seven standards were proposed based on their high resolution MS/MS spectra, which were further used to assist the identification of constituents in extracts. Finally, a total of 131 compounds were detected based on our analytical strategy (Figure 2). Based on their chemical structures, the detected compounds were classified into six groups, including phenylpropanoids, dicaffeoylspermidine/spermine derivatives, phenolic amides, flavones, saponins and others (Figure 2). The detailed information of the 131 compounds found in fruits, leaves and root barks of *L. barbarum* are presented in Table 1.

Phenylpropanoid group included 13 phenylpropionate compounds containing caffeoyl, feruloyl, coumaroyl, sinapoyl or scopoletin group. These groups generate characteristic ions at *m/z* 163 and 145 for caffeoyl in the positive ion mode; *m/z* 163 and 145 for coumaroyl and *m/z* 193 and 175 for feruloyl in the negative ion mode; *m/z* 193/191 and 178/176 for scopoletin in positive/negative ion modes; and at *m/z* 185 and 163 for sinapoyl in negative ion mode. For example, compound A7 with [M + H]^+^ ion at *m/z* 355.1032 (C_16_H_19_O_9_, Cal. 355.1023, mass error 1.55 ppm) was confirmed as chlorogenic acid by a standard. The ion at *m/z* 163.0394 was formed by the neutral loss of a quinoyl unit (193 Da) from the parent ion at *m/z* 355.1032 (Figure S3). Compound A1 displaying a [M − H]^−^ ion at *m/z* 487.1485 (C_23_H_32_O_13_, Cal. 487.1425, mass error 1.78 ppm) was identified to be a lycibarbarphenylpropanoid A isomer; the key product with [M − H − C_12_H_20_O_5_]^−^ ion at *m/z* 163.0403 and [M − H − C_12_H_20_O_5_ − H_2_O]^−^ ion at *m/z* 145.0296 indicated the existence of a coumaroyl moiety (Figure S8). Compound A10 with [M + H]^+^ ion at *m/z* 355.1031 (C_16_H_19_O_9_, Cal. 355.1023, mass error 1.42 ppm) and fragment ions at *m/z* 193.0500 and 178.0276 was identified to be scopolin as confirmed by a standard (Figure S4).

Dicaffeoylspermidine and dicaffeoylspermine derivatives are conjugates of caffeoyl groups and spermidine or spermine via amide bonds, which mainly contain characteristic fragments at *m/z* 310/308, 293/291, 222/220 or 165/163. Compound B9 displaying a [M + H]^+^ ion at *m/z* 531.3220 (C_28_H_43_O_6_N_4_, Cal. 531.3177, mass error 1.41 ppm) was confirmed to be the dicaffeoylspermine derivative kukoamine B by a standard (Figure S5). The fragment ion at *m/z* 367.2736 was formed by neutral loss of one caffeoyl unit, and the fragment ion at *m/z* 165.0559 was formed by further neutral loss of the spermine unit. In the same manner, compounds B1, B5, B7 and B10 were identified to be isomers of kukoamine B.

Compounds B2 and B3 displaying [M + H]^+^ ions at *m/z* 855.4222 (C_40_H_62_O_16_N_4_, Cal. 855.4231, mass error 1.38 ppm) were identified to be two positional isomers of diglycosyl-caffeoyl spermine. Compounds B52 and B63 displaying [M + H]^+^ ion at *m/z* 474.2585 (C_25_H_35_O_6_N_3_, Cal. 474.2598, mass error 2.78 ppm) and 472.2389 (C_25_H_33_O_6_N_3_, Cal. 472.2319, mass error 1.64 ppm) were dicaffeoylspermidines. Their fragment ions at *m/z* 310.2120 and 310.2119 were formed by neutral loss of one caffeoyl unit, and *m/z* 165.0560 and 163.0389 were formed by further neutral loss of the spermidine unit (Figures S9 and S10).

Compounds B18, B31, B42, B48 and B55 displaying [M + H]^+^ ions at *m/z* 634.2959 (C_31_H_44_O_11_N_3_, Cal. 634.2970, mass error 1.31 ppm) and fragment ions at *m/z* 310, 220 and 163 were identified to be five positional isomers of diglycosyl-caffeoyl spermidine. Their common fragment ion at *m/z* 472 is formed by the cleavage of one glucosyl (162 Da, Figure S11). Zhou et al. reported 15 dicaffeoylspermidine derivatives in the fruit of *L. barbarum* [32]. Here, we further found more dicaffeoylspermidine and dicaffeoylspermine derivatives; for example, compounds B46, B50 and B54 found in fruit of *L. barbarum* showing similar [M + H]^+^ ions and fragment ions (Figure S12) were tentatively identified to be isomers of dicaffeoylspermidine derivative with four glucosyls. Compound B46 readily yielded a strong [M + H]^+^ ion at *m/z* 1120.4628 (C_49_H_74_O_26_N_3_, Cal. 1120.4555, mass error 1.58 ppm) and main fragment ions at *m/z* 958.4113 [M + H − C_6_H_10_O_5_]^+^, *m/z* 796.3885 [M + H − C_6_H_10_O_5_ − C_6_H_10_O_5_]^+^, *m/z* 634.2957 [M + H − C_6_H_10_O_5_ − C_6_H_10_O_5_ − C_6_H_10_O_5_]^+^, *m/z* 472.2471 [M + H − C_6_H_10_O_5_ − C_6_H_10_O_5_ − C_6_H_10_O_5_ − C_6_H_10_O_5_]^+^, corresponding to successive losses of glucosyl units. Similarly, compound B61 that yielded a strong [M + H]^+^ ion at *m/z* 1118.4481 (C_49_H_72_O_26_N_3_, Cal. 1118.4396, mass error 1.342 ppm) also contains four hexoses (Figure S13). 

The detected phenolic amides mainly consist of feruloyl, caffeoyl or coumaroyl groups and different amino groups; they generated fragment ions at *m/z* 177, 163 and 147, respectively. Many of them contained a tyramine moiety (137 Da), which lost a NH_3_ to generate a vinylphenol ion at *m/z* 121. Compound C15 displaying a [M + H]^+^ at *m/z* 284.1274 (C_17_H_18_O_3_N, Cal. 284.1281, mass error 1.29 ppm) was identified to be *N*-*p-trans*-coumaroyltyramine and confirmed by a standard, which generated fragment ions at *m/z* 147 and 121 (Figure S6). The ion at *m/z* 147.0445 was formed by the neutral loss of a tyramine unit. Compound C15 displaying a [M + H]^+^ at *m/z* 314.1382 (C_18_H_20_O_4_N, Cal. 314.1387, mass error 1.67 ppm) was identified to be *N*-feruloyltyramine; the key product ions at *m/z* 177.0550 and 121.0658 indicate the existence of feruloyl and tyramine moieties (Figure S14). 

Flavones included six compounds containing quercetin and kaempferol groups, respectively. These groups generated characteristic ions at *m/z* 303 (quercetin), 287 (kaempferol) and a series of fragment ions that continuously lost a CO (28 Da) or CO_2_ (44 Da). Compound D5 displaying a [M + H]^+^ ion at *m/z* 611.1600 (C_27_H_31_O_16_, Cal. 611.1607, mass error 1.03 ppm) and fragment ions at *m/z* 303.0492, 285.0413, 257.046, 201.0561 was identified to be rutin and confirmed by a rutin standard (Figure S7). The fragment ions at *m/z* 465.1061 [M + H − C_6_H_10_O_4_]^+^, 303.0492 [M+H − C_6_H_10_O_4_ − C_6_H_10_O_5_]^+^, 285.0413 [M + H − C_6_H_10_O_4_ − C_6_H_10_O_5_ − H_2_O]^+^, correspond to successive losses of rhamnosyl, glucosyl and H_2_O. The fragment ion at *m/z* 201.05612 was formed by the successive loss of three CO units from the ion at *m/z* 285.0413. In the same manner, compound D6 displaying a [M + H]^+^ ion at *m/z* 595.1703 (C_27_H_31_O_15_, Cal. 595.1657, mass error 0.59 ppm) and key product ions at *m/z* 287.0529, 258.2196, 243.5895 and 230.3383 was identified to be kaempferol-O-Glu-O-Rha. The fragment ions at *m/z* 449.1408 [M + H − C_6_H_10_O_4_]^+^, 287.0528 [M + H − C_6_H_10_O_4_ − C_6_H_10_O_5_]^+^, correspond to the loss of the rhamnosyl and glucosyl units (Figure S15). 

Saponins contain a steroid skeleton and generate key fragment ions at *m/z* 273/271 and 255/253 depending on the presence of a single bond or a double bond at the positions 5 and 6 (Figure 1 and Figure S2). Compounds E2 and E4 were identified to be solasonine and 5,6-dihydrosolasonine by the standards and fragment ions as described above. Compound E3 was tentatively identified to be parillin with [M + H]^+^ at *m/z* 1049.5527 (C_45_H_76_O_16_N, Cal. 1049.5527, mass error 0.90 ppm), and fragment ions at *m/z* 887.4990 [M + H − C_6_H_10_O_5_]^+^, 743.3850 [M + H − C_6_H_10_O_5_ − C_6_H_10_O_4_]^+^, 579.3932 [M + H − C_6_H_10_O_5_ − C_6_H_10_O _4_ − C_6_H_10_O_4_]^+^, 417.3392 [M + H − C_6_H_10_O_5_ − C_6_H_10_O_4_ − C_6_H_10_O_4_ − C_6_H_10_O_5_]^+^, 273.2232 and 255.2125 (Figure S16). It is worth mentioning that four of the six identified saponins, E1 (gracillin), E2 (solasonine), E4 (5,6-dihydrosolasonine) and E6 (lycioside B) have the same sugar moiety, solatriose, but other steroidal glycoalkaloids from *Solanum* plants consist of the same aglycones and different sugar moieties, such as solamargine (chacotriose + solasodine) and soladulcine A/B (chacotriose/lycotetraose + soladulcidine), which were not detected in the three parts of *L. barbarum,* suggesting that solatriose might be a characteristic sugar unit in *L. barbarum*. Saponins are rarely reported in *L. barbarum* [1,47]. All the saponins (E1–E6) reported here were found in *L. barbarum* for the first time. 

Eleven other compounds were also detected, but their diagnostic ions were not included in the characteristic ion database. Consequently, these compounds were not classified. 

**Figure 3:**
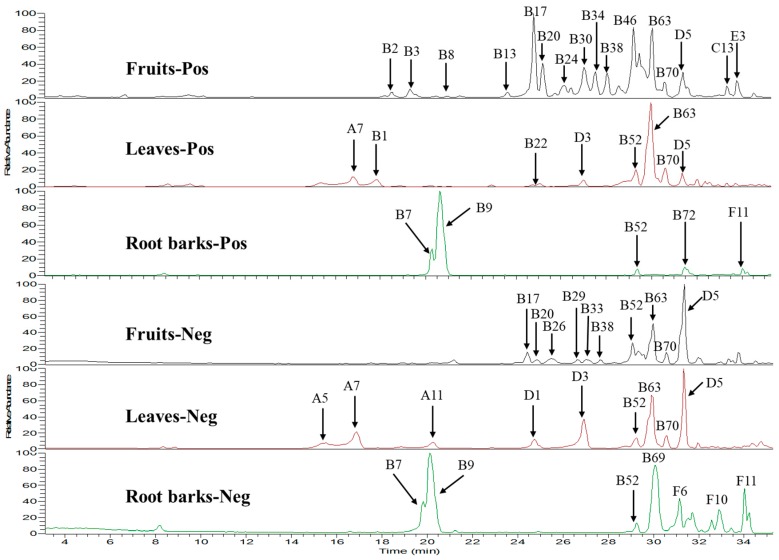
Total ion chromatograms (TICs) of extracts of fruits, leaves and root barks in positive and negative ion mode using UPLC-HR-MS. All the compound numbers were same with those shown in Table 1.

In summary, a total of 131 compounds including 13 phenylpropanoids, 74 dicaffeoylspermidine or dicaffeoylspermine derivatives, 21 phenolic amides, six flavonoids, six saponins and 11 others were detected. Among them, 98 compounds were found in fruits, 28 compounds were found in leaves and 35 compounds were found in root barks of *L. barbarum*, suggesting that the fruits extract contains many more components than root barks and leaves ones. As shown in the total ion chromatograms of UPLC-HR-MS (Figure 3), the distribution of these constituents in the extracts of fruits, root barks and leaves are obviously different. A lot of dicaffeoylspermidine or dicaffeoylspermine derivatives were detected in fruits, leaves and root barks. Among them, kukoamine A (B7) and kukoamine B (B9) (dicaffeoylspermine isomers, main peaks around 20 min in positive/negative modes) and propionyl-dicaffeoylspermidine (B69, main peak around 30 min in negative mode) were found in the extract of root barks in a very large amount, but not detected in leaves and fruits. Dicaffeoylspermidine isomers (B52, B63 and B70) were detected in a high amount in fruits and leaves, but were much lower in root barks. However, some glycosylated derivatives of kukoamine A/B isomers (such as B2, B3, B4, B6, B8, B66 and B72) and many glycosylated derivatives of dicaffeoylspermidine (such as B13-20, B24-42, B30, B38, B44, B46-51, B53-62, B64 and B67) were mainly found in fruits, suggesting that dicaffeoylspermidines and dicaffeoylspermines were glycosylated in the fruit of *L. barbarum*. The difference of the distribution of dicaffeoylspermine and dicaffeoylspermidine derivatives suggests low chemical similarity between root barks and fruits/leaves. Based on Table 1, phenylpropanoids were mainly found in the fruits and leaves, but not detected in the root barks. Phenolic amides were found in all the three parts of *L. barbarum* plants. Flavonoids were mainly found in the fruits and leaves in a high amount, e.g., rutin (D5). Saponins were only detected in the fruits of *L. barbarum*. 

### 2.3. Quantitative Analysis of Seven Compounds in the Fruits, Leaves and Root Barks

The quantitative analysis of seven compounds in the extracts was performed by UPLC-Qtrap-MS with MRM mode owing to its high sensitivity, specificity, and selectivity in the quantitation of trace compounds in complex matrices [18]. The method validation data are listed in Table S2. The calibration curves of the seven standards showed good linearity (r from 0.989 to 0.998). The limit of detection (LOD) and the limit of quantification (LOQ) for each standard were 0.08–0.26 µg/mL and 0.02–0.07 µg/mL. Both intra-day (*n* = 3) and inter-day (*n* = 6) precision was evaluated and the RSDs of the seven standards were less than 4.57% and 3.86%. The recoveries obtained in this study were in the range of 92–112% (Table S3) with low RSDs (<7%) of all standards, demonstrating that the analytical method developed has high accuracy and good reproducibility.

The quantitative results were shown in Table 2. Kukoamine B was only detected in the root barks in a very high amount (10.9 mg/g dry powder). The amount of rutin (D5) in leaves (663.45 µg/g dry leaves) was much higher than that in fruits (93 μg/g dry fruits). However, it was not found in root barks, which was consistent with the results of the above qualitative assay (Figure 3). Chlorogenic acid was only observed in leaves in a high amount (1577 μg/g dry leaves). *N*-*p-trans*-coumaroyltyramine was found in both fruits and leaves in a little amount (<15 μg/g dry powder). Dihydrosolasonine was only found in fruits in an amount of 43 µg/g dry fruit. The major steroidal glycoalkaloid in Solanaceae, solasonine, was only found in fruits in a very low amount (2.16 µg/g dry fruits), which is much lower than that found in *Solanum xanthocarpum* (800 µg/g) [48].

### 2.4. Antioxidative Activity Assays

Since a lot of phenolic compounds (phenylpropanoids, dicaffeoylspermidine/dicaffeoyl-spermine derivatives, phenolic amides, flavonoids) were found in the extracts, their antioxidative activities were compared using 2,2-diphenyl-1-picrylhydrazyl (DPPH), 2,2’-azinobis-(3-ethyl-benzthiazoline-6-sulphonate) (ABTS) and ferric reducing ability of plasma (FRAP) assays [49,50]. The results demonstrated that all the extracts showed antioxidative activities (Table 3). Both DPPH and ABTS assays showed that root bark extract possessed the strongest free radical-scavenging capacity, leaf extract was the second, and fruit extract showed much lower free radical-scavenging capacity than either leaf or root bark extracts. The FRAP assay also showed that the fruit extract had the weakest reducing ability. The strong antioxidative activity of the root bark extract could be explained by the huge amount of kukoamine A/B and propionyl-dicaffeoylspermidine in the root barks [51]. The higher amount of chlorogenic acid and rutin in leaves than those in fruits might explain the higher antioxidative activity of the leaf extract. The overall much higher antioxidant ability of leave and root bark extracts than that of the fruit extract is different from our common understanding that the fruits have strong antioxidant ability.

### 2.5. Protective Effects of Extracts on H_2_O_2_-Induced Oxidative Stress in Cells

**Figure 4:**
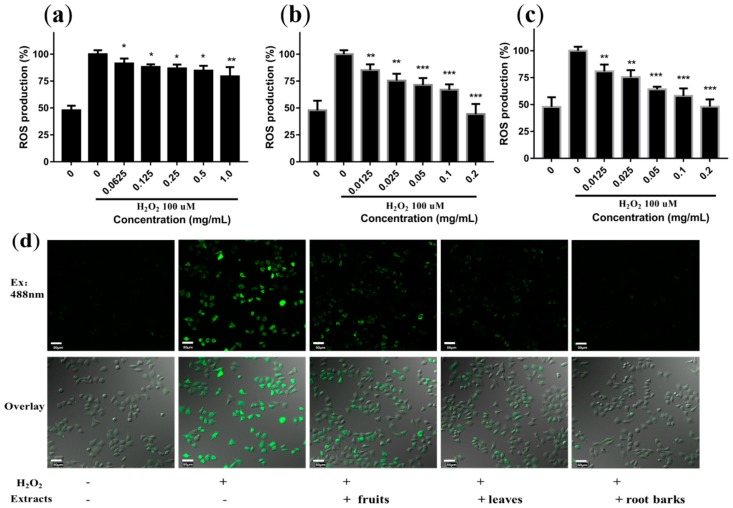
Protection effect of different concentrations of extracts from fruits (**a**), leaves (**b**) and root barks (**c**) on 100 µM H_2_O_2_-induced intracellular ROS production in L02 cells. Results were expressed as means ± SD, *n* = 3. * *p* < 0.05, ** *p* < 0.01, *** *p* < 0.001, vs. the H_2_O_2_ treated group. (**d**) Confocal imaging of intracellular ROS levels after H_2_O_2_ stimulation in the presence of extracts of fruits (1 mg/mL), leaves and root barks (0.2 mg/mL) (scale bar, 50 μm).

H_2_O_2_ triggers oxidative damage through an increase of intracellular ROS. Therefore, the effects of extracts on the production of ROS in H_2_O_2_-exposed L02 cells were measured by 2’,7’-dichloro-dihydrofluorescein diacetate (DCFHDA) assay (Figure 4). ROS in cells can oxidize DCFH (no fluorescence) to form high fluorescent DCF. The results revealed that all the three extracts caused a dose-dependent attenuation of the H_2_O_2_-induced ROS production in L02 cells. The leaf and root bark extracts showed significantly higher antioxidative activity than that of fruit extract (Figure 4a–c and Figure S17).

The ROS in cells treated by 0.25 mg/mL fruit extract was only reduced to 86%, while the ROS in cells treated by 0.2 mg/mL leaf and root extracts were reduced to 44% and 48% respectively. The confocal imaging of cells (Figure 4d) also showed that the three extracts greatly reduced the fluorescence intensity in cells, and the fruit extract showed the weakest effect. This set of results was consistent with the results of the above antioxidative activity analysis in solutions.

### 2.6. In Vitro Assays of Cytotoxicity

**Figure 5:**
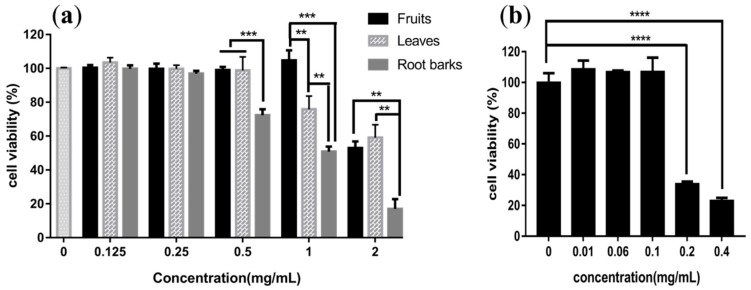
The cytotoxicities of different concentrations of extracts from fruits, leaves, root barks (**a**) and kukoamine B (**b**) towards L02 cells. Results were expressed as means ± S.D., *n* = 3. ** *p* < 0.01, *** *p* < 0.001, **** *p* < 0.0001.

Glycoalkaloids are reported to have anticarcinogenic activity because of their cytotoxicity (IC_50_ = 50 µg/mL) [52]. Thus, the cytotoxicities of the fruit, leaf and root bark extracts to L02 cells were measured by a CCK-8 assay. L02 cell line was derived from the human hepatic and have been widely used in the evaluation of basic cytotoxicity profiles of drug candidates. As shown in Figure 5, the fruit extract did not show cytotoxicity at 1 mg/mL but showed cytotoxicity at 2 mg/mL. The leaf extract showed weak cytotoxicity at 1 mg/mL. However, the root bark extract showed weak cytotoxicity at 0.5 mg/mL, and strong cytotoxicity at 2 mg/mL. The strongest cytotoxicity of the root bark extract may come from the very large amount of dicaffeoylspermidines (kukoamine A/B and propionyl-dicaffeoylspermidine). To prove this hypothesis, the cytotoxicity of kukoamine B was tested. The results showed that kukoamine B (Figure 5b) had strong toxicity at 0.2 mg/mL, which was equivalent to the level of kukoamine B in ~6 mg/mL root bark extract. Considering the similar amount of kukoamine A and propionyl-dicaffeoylspermidine existed together with kukoamine B in root bark extract, the higher cytotoxicity of root bark extract could mainly due to the high content of dicaffeoylspermidine/spermine derivatives. Although glycoalkaloids were only detected in the fruit extract, the extremely low levels cannot cause cytotoxicity. 

### 2.7. Potential Active Compounds Assay in Cells

Since the fruit of *L. barbarum* is mostly used as a functional food and medicinal source, and contains much more components than leaves and root barks, the potential active compounds assay in the extracts will provide helpful information for understanding the action mechanism of fruits of *L. barbarum*. In general, to exert an effect, bioactive molecules should bind receptors or enzymes on cell membranes or enter into cells to interact with their molecular targets. Although a large number of compounds are presented in the extract of plant herbs, only a few of them can bind or enter into cells. Therefore, the cell-based screening has been applied for identification of potential bioactive components in plant herbs [53]. In order to identify the potential active compounds, a compound database of fruits of *L. barbarum* containing 91 chemicals was successfully established based on the quasimolecular ions in Q1 and the characteristic fragment ions in Q3 as ion pairs in MRM mode for the first time. The MRM ion pairs and corresponding declustering potential (DP) and collision energy (CE) of each constituent were optimized and presented in Table S4. 

**Figure 6:**
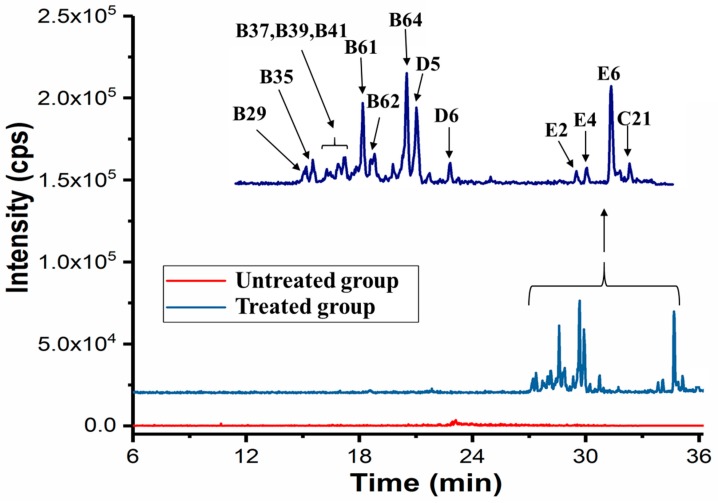
TIC of the extract of L02 cells treated and untreated with fruit extract. All the compound numbers were same with those shown in Table 1 and Table S4.

To screen bioactive molecules, L02 cells were incubated with the fruit extracts for 4 h and then extracted with methanol. The methanol extract of cells was analyzed by UPLC-Qtrap-MS in MRM mode under the same condition with the construction of database. The typical total ion chromatography (TIC) of the extracts of L02 cells treated and untreated with fruit extracts are shown in Figure 6. More than 14 compounds were detected with the retention time in the range of 27–36 min, suggesting that the compounds entered cells have relatively high hydrophobicity. In total, at least eight dicaffeoylspermidine derivatives (e.g., B31 and B37) were detected in the cells. Two flavones (rutin (D5) and D6) were observed in L02 cells. Interestingly, three saponins (E2, E4 and E6) were detected in cells with higher relative content compared to that detected in the extract. Because E2 (solasonine) and E4 (5,6-dihydrosolasonine) were detected in the extract in a very low concentration, their observation in cells suggests that solasonine and 5,6-dihydrosolasonine were enriched in cells. These compounds detected in cells may have potential biological activities, which should be clarified in the future research.

## 3. Materials and Methods

### 3.1. Materials and Reagents

The dried fruits, leaves and root barks of *L. barbarum* were purchased from Ningxia Province. Rutin, kukoamine B, chlorogenic acid, scopolin, solasonine and *N*-*p-trans*-coumaroyltyramine were purchased from Baoji Herbest Bio-Tech Co., Ltd. (Baoji, China). 5,6-Dihydrosolasonine were isolated from the fruit of *L. barbarum* and identified by HR-ESI-MS and NMR techniques. Purities of all compounds were above 96% by HPLC analysis. HPLC grade methanol, acetonitrile, and MS grade formic acid were purchased from Thermo Fisher Scientific (Pittsburgh, PA, USA). Other chemicals and solvents were of analytical reagent grade.

L02 cells (normal human hepatic cell line) were obtained from National Experimental Cell Resource Sharing Platform (Beijing, China). Gentamicin, fetal bovine serum (FBS) were acquired from Gibco (Invitrogen, Paisley, UK). Dimethyl sulphoxide (DMSO) was purchased from Panreac (Barcelona, Spain). Dulbecco’s modified Eagle’s medium (DMEM), 2,2-diphenyl-1-picryl-hydrazyl (DPPH), hydrogen peroxide (30% *w*/*w*), 2,4,6-tris(2-pyridyl)-1,3,5-triazine (TPTZ), 2′,7′-dichlorodihydrofluorescein diacetate (DCFH-DA), ascorbic acid and 6-hydroxy-2,5,7,8-tetramethylchroman-2-carboxylic acid (Trolox) were obtained from Sigma-Aldrich (St. Louis, MO, USA).

### 3.2. Sample Preparation

For bioactivity analysis, the fruits, leaves and root barks (50 g of each) of *L. barbarum* were pulverized into powder and extracted thrice by ultrasound with ethanol/water (70:30, v:v) (500 mL, 1 h; 400 mL, 1 h; 400 mL, 1 h), respectively. After filtration and freeze-drying, the crude products were 20.5 g, 14.3 g, 14.5 g, respectively. The stock solutions of the extracts were prepared by dissolving the freeze-dried extracts in DMSO (200 mg/mL). For UPLC-MS analysis, the powder of fruits, leaves and root barks (100 mg of each) were extracted by ultrasound with 1 mL of ethanol/water (70:30, v:v) for 1 h. After centrifugation, the supernatants were applied for UPLC-MS analysis.

### 3.3. Isolation and Identification of 5,6-dihydrosolasonine from Fruit of L. barbarum

The dried fruit of *L. barbarum* (5 kg) was extracted twice by ultrasound with 25 L of 70% ethanol/water for 1 h each time. After filtration, the ethanol was removed under reduced pressure to yield a concentrated solution. The solution was passed through a macroporous resin column (AB-8) and successively eluted with 0, 20, 80% ethanol/water. Finally, the 80% ethanol/water fraction was concentrated under reduced pressure, then extracted with butanol for three times and concentrated under vacuum. After dissolving this fraction with methanol, the target compounds were purified by preparative high performance liquid chromatography. The HR-ESI-MS data were recorded on a Orbitrap mass spectrometer (Thermo Fisher Scientific, Bremen, Germany). NMR spectra were acquired with Bruker AV 600 spectrometers (Bruker BioSpin Group, Faellanden, Switzerland) using the solvent signals (in C_5_D_5_N) as internal standards.

### 3.4. Multiple Component Identification by UPLC-Orbitrap-MS

The ultimate 3000 hyperbaric LC system coupled with high resolution Orbitrap Fusion Lumos Tribrid^TM^ via an electrospray ionization (ESI) interface (Thermo Fisher Scientific) was used for a comprehensive analysis of the constituents in fruit, leaf and root bark extracts. A BEH C18 column (1.7 μm, 2.1 mm ID × 100 mm, Waters, Milford, MA, USA) maintained at 35 °C was finally chosen for separation of these extracts. The mobile phase was water (0.1% formic acid, A) mixed in gradient mode with acetonitrile (0.1% formic acid, B), at a flow rate of 200 μL/min. The elution gradient was optimized as follows: 0–3 min, 3% B; 3–10 min, 3% to 5% B; 10–25 min, 5% to 20% B; 25–30 min, 20% to 50% B; 30–33 min, 50% to 100% B; 33–42min, 100% B. The injection volume was 3.0 μL and samples were set at 4 °C.

For identification of the components in the extracts, both positive and negative full scan modes within the mass/charge (*m/z*) ratio range of 150–1500 at a resolution of 120,000 were used for acquisition of accurate molecular ions. The other parameters were as follows: spray voltage, + 3.0 kV in the positive mode and − 2.0 kV in the negative mode; sheath gas flow rate, 35 Arb; aux gas flow rate, 10 Arb; sweep gas flow rate, 2 Arb; ion transfer tube temperature, 325 °C, vaporizer temperature, 275 °C. The fragment ions in MS/MS data obtained by higher energy collision dissociation (HCD, collision energy: 35 eV) were further utilized for confirmation of the structures of components. In addition, standards were also used for assistance of component identification. Xcalibur^TM^3.0 software was used for UPLC-HR-MS control and data handling.

### 3.5. Compound Database Construction by UPLC-Qtrap-MS

An AB Sciex Qtrap^®^ 4500 tandem MS (Foster City, CA, USA) equipped with an ESI source connected to the UPLC system (I-class Acquity UPLC, Waters, Framingham, MA, USA) was used to construct the compound database. Firstly, an instrument method in MRM (Q1 = Q3) information-dependent acquisition (IDA)-enhanced product ion (EPI) mode [18] for the analysis of fruit extract was established on the basis of the identified compounds from fruits by UPLC-HR-MS. Further, product ion scanning experiments were conducted and the DP and CE was optimized for each analyte to generate the most abundant product ions. The product ion spectra were further used to select the precursor-product ion pairs for the development of MRM assays. Finally, a compound database of fruit of *L. barbarum* was established based on the quasimolecular ion in Q1 and its characteristic fragment ions in Q3 as ion pair in MRM mode. This database was used for the next screening of active compounds.

### 3.6. Quantitative Analysis of Compounds in Extracts

UPLC-Qtrap-MS was used for quantitative analysis of seven compounds in the extracts of fruits, leaves and root barks by MRM mode. The liquid chromatographic conditions were the same as those of UPLC-Orbitrap-MS analysis. Method validation was carried out for seven standards in terms of linearity, sensitivity, intra/inter-day precisions and recovery [54]. The linearity was obtained by preparing a series of concentrations of standards solution with at least five appropriate concentrations in duplicate. The LOD and the LOQ for each analyte were acquired while the S/N was 3 and 10, respectively. The precision (inter and intra-day precision) was analyzed using the standard solutions with six replicates, and the RSD of the peak area for each standard was calculated. A spike recovery test was used to evaluate the accuracy of these methods. Three concentrations (high, middle and low) of mixed standard solutions were added to fruit extract respectively, then quantitative analysis was performed as described above. Each standard was tested at each concentration in triplicate. The spike recoveries were calculated using the following equation: Recovery% = [(measured amount-original amount)/amount added] × 100, RSD = SD/mean × 100 [55]. Additionally, quantification of seven compounds using UPLC-Qtrap-MS was also established and the MRM pairs, DP and CE were optimized based on the standards.

### 3.7. In Vitro Antioxidative Assays

#### 3.7.1. DPPH Radical Scavenging Activity

The effect of the extracts against 2,2-diphenyl-1-picrylhydrazyl (DPPH) radical was tested according to a previous report [56] with slight modifications. In brief, 100 μL of 0.2 mM DPPH radical solution in ethanol was added to 100 μL of extract solutions at different concentrations. After incubation for 30 min at room temperature in the dark, the absorbance was read at 517 nm. Ascorbic acid was used as a positive control and all measurements were done in triplicate. The extract concentration providing 50% inhibition (IC_50_) was calculated by plotting inhibition percentages against the concentrations of extracts. The DPPH radical scavenging rate (S%) was calculated as follows: S% = [(A_0_−A_1_)/A_0_] × 100 (A_1_ and A_0_ are the absorbance of DPPH radical solution after incubation with and without extracts, respectively). 

#### 3.7.2. ABTS Radical Scavenging Assay

ABTS^•+^ scavenging activity was measured according to the defined method with slight modifications [57]. In brief, the radical cations were prepared by reacting 7 mM aqueous ABTS with 2.45 mM potassium persulphate. The mixture was allowed to stand in the dark at room temperature for 16 h before use and the ABTS^•+^ solution was diluted with methanol to an absorbance of 0.700 ± 0.020 at 734 nm. Different concentrations of extracts in methanol (40 μL) were added to 160 μL of ABTS^•+^ solution and the absorbance was recorded after 4 min. The IC_50_ and percentage inhibition of absorbance at 734 nm were calculated. All measurements were done in triplicate. Inhibition of ABTS^•+^ in percent, I (%) was calculated as follows: I (%) = (A_b_−A_s_/A_b_) × 100, where A_b_ was the absorbance of the control and A_s_ was the absorbance of tested samples. 

#### 3.7.3. FRAP Assay

The principle of the FRAP assay is based on the reduction of ferric-tripyridyltriazine complex to its ferrous (colored form) in the presence of antioxidants. The FRAP assay was performed as described previously [58]. Briefly, the FRAP reagent contained 5 mL of 10 mM TPTZ (2,4,6-tripyridy-s-triazine) solution in 40 mM HCl and 5 mL of 20 mM FeCl_3_·6H_2_O in 300 mM acetate buffer (pH = 3.6). The mixture was freshly prepared and warmed at 37 °C for 30 min. In parallel, a solution containing 5 μL of ultrapure water and 155 μL of FRAP solution was prepared as the negative control. Different concentrations of extracts in methanol (5 μL) were mixed with 155 μL of FRAP solution and kept for 30 min in the dark. The ferrous tripyridyltriazine complex (coloured product) was measured by reading the absorbance at 593 nm. The antioxidative capacity of test samples was given by the RC_50_ value, the concentration (μg/mL) necessary for a 50% reduction of Fe^3+^. Trolox was used as the positive control with a concentration ranging from 0 to 150 μg/mL.

### 3.8. Reactive Oxygen Species Measurement in L02 Cell

In cells, reactive oxygen species (ROS) were determined using a fluorescent dye protocol [59]. Cells were treated with different concentrations of each extract for 1 h and then incubated with H_2_O_2_ (100 µM) for 1 h. The DCF fluorescence intensity was detected on a SpectraMax M5 microplate reader (Molecular Devices, Sunnyvale, CA, USA) with excitation at 488 nm and emission at 535 nm. The confocal imaging was performed on an OLYMPUS FV3000-IX81 confocal microscope (Olympus Corporation, Tokyo, Japan). Confocal images were processed by Olympus FV10-ASW 4.2 viewer software (Olympus Corporation, Tokyo, Japan).

### 3.9. Cytotoxicity Assay

L02 cells were cultured in 10% FBS-supplemented DMEM and 1% gentamicin, and kept in a humidified atmosphere of 5% CO_2_ at 37 °C. For cytotoxicity assay, L02 cells in logarithmic growth phase were plated in 96-well plates at a density of 5 × 10^3^ cells per well in 100 μL of culture medium and were allowed to adhere for 24 h before treatment. Serial concentrations of each sample (fruit, leaf and root bark extracts and kukoamine B) were then added (100 μL per well). After treated for 24 h, 10 μL of CCK-8 solution was added to each well and incubated at 37 °C, 5% CO_2_ for 1 h. The absorbance at 450 nm was measured using a SpectraMax M5 microplate reader (Molecular Devices).

### 3.10. Target Cell-Based Screening of Potential Active Compounds

The target cell-based screening of potential active compounds in the fruit of *L. barbarum* was performed as described in previous study with slight modifications [15]. Specifically, L02 cells in the logarithmic growth phase were seeded into cell culture dish at a density of 1.0 × 10^6^ cells/mL, and were cultured in DMEM medium at 37 °C for 24 h. The culture medium was replaced by 3 mL of fruits extract of *L. barbarum* diluted in DMEM (free of serum) at a final concentration of 10 mg/mL, and incubated at 37 °C for 4 h. The incubation solution was discarded and the cells were washed five times with phosphate-buffered saline to remove free components. Finally, the cells were collected and extracted with 200 μL of methanol and centrifuged at 12,000 rpm for 10 min. The obtained supernatant was used for UPLC-Qtrap-MS analysis. The control sample without extract treatment was prepared by the same procedures as above.

### 3.11. Data Handling and Presentation/Statistical Analysis

Data for quantification were acquired from individual experiments repeated at least three times, and expressed as the means ± SD. Statistical significance was calculated by GraphPad Prism 6 software (GraphPad Software, Inc., San Diego, CA, USA) with unpaired two-tailed t-tests and accepted by *p* < 0.05 (*), *p* < 0.01 (**), *p* < 0.001 (***), *p* < 0.0001 (****). The IC_50_ or RC_50_ were calculated using the GraphPad Prism 6 software according to the inhibition rates or reduction rates (y) plotted against the sample concentrations (x). 

## 4. Conclusions

In this study, a total of 131 compounds were identified in extracts (70% ethanol) from fruits, leaves and root barks of *L. barbarum* by UPLC-Orbitrap-MS and seven of them were quantified by UPLC-Qtrap-MS. The distribution of these compounds in the three parts of *L. barbarum* was significantly different. The fruit extract contained the most compounds. A very large amount of kukoamine A/B (dicaffeoylspermine isomers) and propionyl-dicaffeoylspermidine were found in the extracts of root barks, and a high amount of dicaffeoylspermidine isomers were detected in the fruits and leaves. Many glycosylated derivatives of dicaffeoylspermine/dicaffeoylspermidine were mainly detected in the fruits. The bioactivity assays showed that the fruit extracts had the lowest antioxidant activity and cytotoxicity. The root bark extracts showed the strongest antioxidant activity and cytotoxicity, which was caused by the large amount of dicaffeoylspermidine/spermine derivatives. Six saponins were found in *L. barbarum* plants for the first time, and they were only detected in the fruits; among them, 5,6-dihydrosolasonine, a new glycoside alkaloid (saponin) was isolated and characterized. In addition, 14 potential bioactive compounds were detected in L02 cells after treated with the extracts of fruits. All these results will provide important information for understanding the different biological activities of the three parts of *L. barbarum* plants and will be beneficial for drug discovery from *L. barbarum* plants.

## Figures and Tables

**Table 1:** Compounds identified from fruits, leaves and root barks of *L. barbarum* by UPLC-Q-Orbitrap-MS/MS.

No	RT ^1^	Formula	[M + H]^+^	[M − H]^−^	ppm	MS/MS Fragments ^2^	Identification	Ref. ^3^	F ^4^	L ^5^	R ^6^
A1	8.70	C_21_H_29_O_13_		487.1485	1.78	324.8671; 303.7701; **163.0403**; 145.0296; 119.0503	Lycibarbarphenylpropanoid A isomer	[28]	✓		
A2	9.72	C_21_H_29_O_13_		487.1492	1.22	398.1759; 229.0514; **163.0381**; 145.0275; 119.0503	Lycibarbarphenylpropanoid A isomer	[28]	✓		
A3	10.44	C_22_H_30_O_14_		517.1583	0.72	334.8634; 235.0619; **193.0510**; 175.0403; 160.0169; 134.0375	Lycibarbarphenylpropanoid C isomer	[28]	✓		
A4	10.47	C_15_H_16_O_8_		325.0937	0.23	298.8872; **163.0398**; 145.0276; 119.0497	p-coumaric acid O-glycosides	[16]	✓	✓	
A5	15.45	C_16_H_18_O_9_	355.1050	353.0854	1.34	308.9027; 285.0117; 181.0513; **163.0402**; 145.0296;	chlorogenic acid isomer	[28]		✓	
A6	16.16	C_22_H_30_O_14_		517.1594	1.58	354.5810; 259.0620; **193.0510**; 175.0403; 160.0169; 134.0375	Lycibarbarphenylpropanoid C isomer	[28]	✓		
A7	16.88	C_16_H_18_O_9_	355.1050	353.0854	1.55	163.0395; 145.0289; 135.0445; **117.0339**; 89.0390;	chlorogenic acid *			✓	
A8	17.32	C_23_H_32_O_13_		515.1417	1.00	395.0988; 353.0877; 274.9858; **191.0557**; 161.0242	Lycibarbarphenylpropanoid F isomer	[28]	✓	✓	
A9	18.78	C_23_H_32_O_13_		515.1422	1.24	323.0873; 274.9858; **191.0539**; 161.0225	Lycibarbarphenylpropanoid F isomer	[28]	✓	✓	
A10	18.87	C_16_H_18_O_9_	355.1008	353.0855	1.42	303.0228; **193.0512**; 178.0276; 134.0375	Scopolin *		✓		
A11	18.92	C_27_H_36_O_18_		647.1873	1.34	485.2170; 323.1666; **191.0319**; 176.0087; 161.0431; 148.0142	lycibarbarcoumarin A	[28]	✓		
A12	20.42	C_16_H_18_O_9_	355.1047	353.0854	1.34	285.0116; 193.0510; **163.0403**; 145.0295; 123.1177	chlorogenic acid isomer	[28]		✓	
A13	30.04	C_17_H_22_O_10_		385.1140	1.29	326.9597; 185.0199; **163.0381**; 119.0486	sinapate 4-*O*-β-glucopyranoside	[29]	✓	✓	
B1	17.87	C_28_H_42_O_6_N_4_	531.3170	529.3001	1.41	513.3074; 367.2724; **293.1855**; 222.1123; 165.0546	kukoamine B ismoer	[30]		✓	
B2	18.48	C_40_H_62_O_16_N_4_	855.4222	853.4034	1.38	693.3734; 529.3273; **455.2405**; 384.1668; 293.1871; 222.1121; 165.0545	2Glu-[kukoamine] ismoer		✓		
B3	19.30	C_40_H_62_O_16_N_4_	855.4222	853.4034	1.38	693.3693; 531.3206; **455.2379**; 384.1668; 293.1853; 222.1121; 165.0545	2Glu-[kukoamine] ismoer		✓		
B4	19.48	C_46_H_72_O_21_N_4_	1017.4749	1015.4559	1.31	855.4216; 617.2904; **455.2379**; 384.1645; 222.1122; 165.0557	3Glu-[kukoamine] ismoer		✓		
B5	19.50	C_28_H_42_O_6_N_4_	531.3213	529.3011	1.41	513.3074; 367.2728; **293.1855**; 222.1123; 165.0546	kukoamine B ismoer		✓		
B6	19.59	C_34_H_52_O_11_N_4_	693.3698	691.3521	1.05	531.3168; 367.2730; 293.1855; **222.1122**; 165.0560; 123.0440	Glu-[kukoamine] ismoer		✓		
B7	20.28	C_28_H_42_O_6_N_4_	531.3165	529.3001	1.41	513.3103; 447.8046; 376.2692; **293.1879**; 222.1140; 165.8764	kukoamine A	[30]			✓
B8	20.60	C_46_H_72_O_21_N_4_	1017.4747	1015.4559	1.49	855.4216; 617.2901; **455.2379**; 384.1642; 222.1121; 165.0552	3Glu-[kukoamine] ismoer		✓		
B9	20.65	C_28_H_42_O_6_N_4_	531.3220	529.3001	1.41	513.3074; 367.2712; 310.2152; **293.1855**; 222.1123; 165.0545	kukoamine B *				✓
B10	21.02	C_28_H_42_O_6_N_4_	531.3199	529.3003	1. 40	402.9773; 367.2716; **293.1880**; 222.1141; 193.0510; 165.0553; 129.1395	kukoamine B ismoer	[30]	✓		
B11	21.73	C_28_H_40_O_6_N_4_	529.3059	527.2831	1.13	472.2346; 367.2725; **293.1877**; 222.1140; 163.0402	Dihydrocaffeoyl quinonespermine ismoer ^7^	[31]			✓
B12	23.31	C_28_H_40_O_6_N_4_	529.3012	527.2847	1.72	511.2894; 455.2384; 384.1648; **293.1855**; 220.0986; 163.0406	Dihydrocaffeoyl quinonespermine ismoer	[31]	✓	✓	
B13	23.83	C_43_H_63_O_21_N_3_	958.4016	956.3817	1.18	796.3486; 634.2960; 472.2396; 310.2119; **220.0966**; 163.0388	Glu-[lycibarbarspermidine F] isomer		✓		
B14	24.41	C_37_H_51_O_16_N_3_	794.3339	792.3145	0.36	632.2804; 470.2540; 382.1489; **220.0965**; 163.0388	[lycibarbarspermidine O] isomer	[32]	✓		
B15	24.41	C_43_H_65_O_21_N_3_	960.4161	958.3975	2.28	798.3622; 636.3112; 474.2588; 384.1645; **222.1122**; 163.0402	Glu-[lycibarbarspermidine M]ismoer		✓		
B16	24.75	C_37_H_55_O_16_N_3_	798.3640	796.3453	1.80	636.3071; 474.2589; 384.1644; **220.0965**; 163.0388	[lycibarbarspermidine M] isomer	[32]	✓		
B17	24.78	C_37_H_53_O_16_N_3_	796.3492	794.3301	180	634.2957; 472.2431; 310.2126; **220.0965**; 163.0398	[lycibarbarspermidine F] isomer	[32]	✓		
B18	24.80	C_31_H_43_O_11_N_3_	634.2959	632.2784	1.31	472.2431; 310.2122; **220.0966**; 163.0390	lycibarbarspermidine B isomer	[32]	✓		
B19	24.94	C_43_H_63_O_21_N_3_	958.4267	956.3817	1.82	796.3481; 634.2957; 472.2414; 310.2119; **220.0964**; 163.0388	Glu-[lycibarbarspermidine F] isomer		✓		
B20	25.17	C_37_H_55_O_16_N_3_	798.3640	796.3453	1.89	636.3117; 474.2589; 384.1644; **222.1121**; 165.0545	[lycibarbarspermidine M] isomer	[32]	✓		
B21	25.62	C_28_H_42_O_5_N_4_	515.3267		7.61	498.2995; 367.2717; **293.7878**; 277.1928; 222.1139; 165.0556; 129.1395	Dihydrocaffeoyl spermine derivative				✓
B22	25.72	C_28_H_40_O_6_N_4_	529.3054	527.2847	7.16	458.2317; 367.2724; **291.1721**; 220.0988; 163.0401	Dihydrocaffeoyl quinonespermine ismoer	[31]		✓	✓
B23	25.72	C_28_H_40_O_6_N_4_	529.3059	527.2847	1.72	511.2894; 393.2533; 384.1649; **291.1745**; 220.2846; 163.0422	Dihydrocaffeoyl quinonespermine ismoer	[31]		✓	
B24	25.77	C_43_H_63_O_21_N_3_	958.4016	956.3817	1.69	796.3489; **634.2968**; 472.2392; 310.2118; 220.09656; 163.0383	Glu-[lycibarbarspermidine F] isomer		✓		
B25	26.02	C_37_H_55_O_16_N_3_	798.3626	796.3453	3.63	636.3112; 474.2581; 384.1645; **222.1128**; 165.0544	[lycibarbarspermidine M] isomer	[32]	✓		
B26	26.10	C_37_H_53_O_16_N_3_	796.3409	794.3301	1.92	634.2957; 472.2431; 310.2121; **220.0965**; **163.0390**	[lycibarbarspermidine F] isomer	[32]	✓		
B27	26.36	C_43_H_65_O_21_N_3_	960.4125	958.3969	3.30	798.3621; 636.3112; 474.2590; 384.1646; **222.1121**; 163.0404	Glu-[lycibarbarspermidine M]ismoer		✓		
B28	26.41	C_43_H_63_O_21_N_3_	958.4011	956.3817	1.63	796.3483; 634.2957; 472.2428; 398.1824; 310.2119; **220.0965**; 163.0388	Glu-[lycibarbarspermidine F] isomer		✓		
B29	26.44	C_37_H_55_O_16_N_3_	798.3609	796.3453	1.58	636.3071; 474.2585; 384.16455; **222.1122**; 163.0385	[lycibarbarspermidine M] isomer		✓		
B30	26.79	C_43_H_63_O_21_N_3_	958.4009	956.3817	1.82	796.3530; 634.2994; 472.2431; 382.1511; 310.2120; 220.0978; **163.0398**	Glu-[lycibarbarspermidine F] isomer		✓		
B31	27.02	C_31_H_43_O_11_N_3_	634.2964	632.2784	1.03	472.24500; 310.21201; **220.09660**; 163.03981	lycibarbarspermidine B isomer	[32]	✓		
B32	27.03	C_31_H_41_O_11_N_3_	632.2814	630.2632	0.07	470.2284; 382.1489; 308.1962; **220.0965**; 163.0388	lycibarbarspermidine N isomer	[32]	✓		
B33	27.23	C_43_H_63_O_21_N_3_	958.3870	956.3817	1.80	796.34717; 634.29457; 472.2424; 310.2124; **220.0964**; 163.0383	Glu-[lycibarbarspermidine F] isomer		✓		
B34	27.42	C_43_H_65_O_21_N_3_	960.4155	958.3969	9.00	798.3629; 636.3112; 474.2588; 384.1645; **222.1121**; 165.0404	Glu-[lycibarbarspermidine M] ismoer		✓		
B35	27.51	C_31_H_45_O_11_N_3_	636.3115	634.2941	2.00	474.2589; 384.1649; **222.1121**; 165.0544	lycibarbarspermidine J		✓		
B36	27.73	C_31_H_41_O_11_N_3_	632.2811	630.2632	0.50	470.2276; 382.1480; 308.1962; **220.0963**; 163.0388	lycibarbarspermidine N isomer		✓		
B37	27.85	C_37_H_51_O_16_N_3_	794.3337	792.3145	0.67	632.28010; 470.2540; 382.1489; 220.0965; **163.0388**	[lycibarbarspermidine O] isomer		✓		
B38	27.91	C_43_H_63_O_21_N_3_	958.3870	956.3817	1.22	796.3475; **634.2955**; 472.2420; 310.2120; 220.09654; 163.0388	Glu-[lycibarbarspermidine F] isomer		✓		
B39	28.04	C_37_H_53_O_16_N_3_	796.3495	794.3301	1.39	634.2959; 472.2431; 310.2122; 220.0966; **163.0391**	[lycibarbarspermidine F] isomer	[32]	✓		
B40	28.16	C_43_H_61_O_21_N_3_	956.3857	954.3660	1.48	794.3328; 632.2972; 470.2267; 220.0965; **163.0387**	Glu-[lycibarbarspermidine O] ismoer		✓		
B41	28.30	C_43_H_65_O_21_N_3_	960.4146	958.3969	3.39	798.3626; 636.3109; **474.2587**; 384.1645; 222.1122; 165.0404	Glu-[lycibarbarspermidine M]ismoer		✓		
B42	28.57	C_31_H_43_O_11_N_3_	634.2960	632.2784	1.69	472.2424; 382.1488; 310.2118; 220.0969; **163.0388**	lycibarbarspermidine B isomer	[32]	✓		
B43	28.64	C_29_H_44_O_6_N_4_	545.3377	543.3162	7.85	527.3255; 432.0255; 322.2652; **293.1878**; 236.1295; 222.1139; 129.1396	Dihydrocaffeoyl spermine derivative				✓
B44	28.77	C_41_H_57_O_20_N_3_	912.3596	910.3403	1.38	750.3066; 634.2957; 472.2431; 310.2121; **220.0965**; 163.0398	Dihydrocaffeoyl spermidine derivative		✓		
B45	28.83	C_29_H_44_O_6_N_4_	545.3377	543.3162	7.97	527.3264; 381.2884; **307.4034**; 293.1878; 222.1139; 165.14386; 129.1396	Dihydrocaffeoyl spermine derivative				✓
B46	29.06	C_49_H_73_O_26_N_3_	1120.4537	1118.4336	1.58	958.3557; 796.3481; 634.2957; 310.2118; **220.0965**; **163.0402**	2Glu-[lycibarbarspermidine F] ismoer		✓		
B47	29.11	C_41_H_57_O_20_N_3_	912.3597	910.3403	1.25	750.3068; 634.2960; 472.2431; 310.2121; **220.0965**; 163.0398	Dihydrocaffeoyl spermidine derivative		✓		
B48	29.16	C_31_H_43_O_11_N_3_	634.2959	632.2780	1.79	617.2727; 558.1018; 472.2414; 310.2120; **220.0966**; 163.0388	lycibarbarspermidine B isomer	[32]	✓	✓	
B49	29.23	C_31_H_41_O_11_N_3_	632.2813	630.2630	0.12	604.2890; 587.2619; 470.2543; 382.1489; 308.1963; **220.0965**; 163.0388	lycibarbarspermidine N isomer		✓		
B50	29.29	C_49_H_73_O_26_N_3_	1120.4536	1118.4336	1.69	958.3558; 796.34814; 634.2957; 472.2396; 310.2118; **220.0965**; **163.0412**	2Glu-[lycibarbarspermidine F] ismoer		✓		
B51	29.32	C_37_H_51_O_16_N_3_	794.3336	792.3145	0.82	632.2830; 470.2540; 382.1489; 220.0966; **163.0389**	[lycibarbarspermidine O] isomer		✓		
B52	29.40	C_25_H_35_O_6_N_3_	474.2585	472.2435	2.79	457.2319; 310.2120; **222.1121**; 165.0544; 123.0438	*N*^1^-*N*^10^dihydrocaffeoyl spermidine	[31,33]	✓	✓	✓
B53	29.44	C_41_H_57_O_20_N_3_	912.3593	910.3403	1.71	750.3066; 634.2957; 498.1598; 310.2121; **220.0965**; 163.0398	Dihydrocaffeoyl spermidine derivative		✓		
B54	29.58	C_49_H_73_O_26_N_3_	1120.4535	1118.4336	1.80	958.3557; 796.3481; 634.2957; 310.2115; **220.0965**; **163.0412**	2Glu-[lycibarbarspermidine F] ismoer		✓		
B55	29.59	C_31_H_43_O_11_N_3_	634.2960	632.2784	1.70	513.0655; 472.2426; 310.2116; **222.1120**; 163.0388	lycibarbarspermidine B isomer		✓		
B56	29.60	C_31_H_41_O_11_N_3_	632.2805	630.2630	1.42	496.2304; 470.2259; 382.1489; 308.1962; **220.0965**; 163.0388	lycibarbarspermidine N isomer		✓		
B57	29.64	C_41_H_57_O_20_N_3_	912.3593	910.3403	1.71	750.3065; 588.2751; 382.1488; 310.2121; **220.0964**; 163.0398	Dihydrocaffeoyl spermidine derivative		✓		
B58	29.71	C_37_H_53_O_16_N_3_	796.3480	794.3300	2.31	634.2957; 472.2431; 310.2121; **220.0965**; 163.0390	[lycibarbarspermidine F] isomer		✓		
B59	29.71	C_37_H_51_O_16_N_3_	794.3331	792.3145	1.44	632.2804; 470.2540; 308.1962; **220.0964**; 163.0388	[lycibarbarspermidine O] isomer		✓		
B60	29.74	C_35_H_47_O_15_N_3_	750.3065	748.2889	1.11	588.2747; 472.2429; 310.2117; **220.0964**; 163.0387	Glu-[lycibarbarspermidine B] ismoer		✓		
B61	29.74	C_49_H_71_O_26_N_3_	1118.4481	1116.4180	1.45	956.3852; 794.3327; 632.3016; 470.2233; 220.0965; **163.0388**	2Glu-[lycibarbarspermidine O] isomer		✓		
B62	29.88	C_35_H_49_O_15_N_3_	750.3069	748.2889	1.48	588.2748; 472.2429; 310.2118; **220.0965**; 163.0389	Glu-[lycibarbarspermidine B] ismoer		✓		
B63	30.03	C_25_H_33_O_6_N_3_	472.2389	470.2264	1.64	455.2168; 310.2119; **220.0965**; 163.0388; 112.1121	*N*^1^–caffeoyl, *N*^3^-dihydrocaffeoyl spermidine	[34]	✓	✓	✓
B64	30.13	C_31_H_41_O_11_N_3_	632.2804	630.2628	1.57	470.2497; 382.1489; 308.1962; 220.0965; **163.0388**	lycibarbarspermidine N isomer		✓		
B65	30.14	C_28_H_41_O_8_N_3_	548.3005		7.11	**530.2393**; 474.2629; 293.1877; 222.1940; 165.0555; 128.1080	Dihydrocaffeoyl spermidine derivative				✓
B66	30.14	C_34_H_28_O_12_N_4_	705.3398		7.98	687.3212; 531.3211; 467.2042; 310.2160; **293.1826**; 222.1139; 165.0559	Dihydrocaffeoyl spermine derivative				✓
B67	30.14	C_37_H_51_O_16_N_3_	794.3336	792.3145	0.76	632.2804; 470.2540; 308.1963; 220.0965; **163.0388**	[lycibarbarspermidine O] isomer		✓		
B68	30.19	C_30_H_46_O_6_N_4_	559.3531		1.89	395.2663; **307.2033**; 236.1295; 222.123; 165.0561	Dihydrocaffeoyl spermine derivative				✓
B69	30.19	C_28_H_40_O_7_N_3_	530.2891	528.2696	1.68	474.2629; 310.2142; 293.1877; **222.1140**; 165.0558	Propionyl-dihydrocaffeoyl spermidine				✓
B70	30.56	C_25_H_31_O_6_N_3_	470.2278	468.2115	1.54	308.1962; 291.1698; 234.1121; **220.0965**; 163.0388	*N*, *N*′-dicaffeoylspermidine	[33]	✓	✓	
B71	30.82	C_34_H_46_O_11_N_4_	687.3308	685.3051	7.59	670.3035; 523.2778; 449.1942; 293.1878; **222.1139**	Dihydrocaffeoyl spermine derivative				✓
B72	31.45	C_37_H_50_O_9_N_4_	695.3701	693.3464	1.28	678.3438; 531.3215; 457.2365; **293.1879**; 222.1140; 165.0559	*N*^1^,*N*^4^,*N*^12^-tris(dihydrocaffeoyl)spermine	[35]			✓
B73	30.71	C_46_H_46_O_18_N_4_	963.4437	961.4240	0.87	945.4429; 621.3273; 455.2382; **384.1644**; 293.1882; 222.1121; 165.0557	Ddihydrocaffeoyl spermine derivative		✓		
B74	31.79	C_30_H_45_O_8_N_3_	576.3314	574.3247	1.45	544.3050; 512.2786; 412.2842; 293.1877; **222.1139**; 165.0558	Ddihydrocaffeoyl spermidine derivative				✓
C1	8.30	C_13_H_18_O_3_N_2_	251.1409	249.1227	1.41	234.0990; **163.0697**; 144.0616; 126.0558; 115.0591	*N*-caffeoylputrescine isomer	[36]	✓	✓	✓
C2	10.26	C_13_H_18_O_3_N_2_	251.1410		1.69	234.1142; **163.0402**; 145.0296; 115.0876	*N*-caffeoylputrescine isomer	[36]	✓	✓	
C3	16.49	C_15_H_22_O_4_N_2_	295.1674		1.20	278.1412; **207.0670**; 175.0409; 147.0453; 129.1394	coumaroyl amide derivative	[37]			✓
C4	23.48	C_15_H_22_O_4_N_2_	295.1673		1.17	278.1405; 222.1142; **207.0666**; 175.0412; 147.0451	coumaroyl amide derivative	[37]			✓
C5	30.22	C_21_H_29_O_9_N	476.1905	474.2481	2.14	314.1381; 222.1119; **177.0545**; 145.0284; 121.0648	*N*-feruloyl-3-*O*-glucopyranosyl-tyramine ismoer	[38]	✓		
C6	30.42	C_32_H_45_O_11_N_3_	648.3118	646.2941	1.32	486.2590; 310.2119; **234.1121**; 177.0544; 145.0291	feruloyl-tyramine derivatives		✓		
C7	31.30	C_17_H_18_O_4_N	302.1412	300.1221	1.46	286.0265; 245.8081; **165.0556**; 138.0924; 121.0657	A4 (caffeoyl-tyramine derivatives)	[32]			✓
C8	31.34	C_14_H_14_O_5_N_2_	486.2587	484.2422	1.07	469.2316; 310.2118; **234.1120**; 177.0544; 145.0283; 121.0649	feruloyl-tyramine derivatives		✓		
C9	31.83	C_21_H_29_O_9_N	476.1905	474.2481	2.07	314.1381; 222.1118; **177.0545**; 145.0284; 121.0648	*N*-feruloyl-4-*O*-glucopyranosyl-tyramine	[38]	✓		
C10	32.09	C_36_H_36_O_8_N_2_	625.2537	623.1578	1.73	462.1902; 351.0856; 293.08033; **201.0544**; 175.0402; 149.0609; 121.0648	Lyciumamide A	[39]	✓		
C11	32.22	C_36_H_49_O_16_N_3_	498.2592	498.2576	1.41	480.2489; 322.2119; **234.1126**; 177.0545; 145.0284	feruloyl-tyramine derivatives		✓		
C12	32.57	C_17_H_19_O_4_N	300.1256	298.1066	0.48	253.8823; **163.0402**; 121.0658	A5 (caffeoyl-tyramine derivatives)	[32]			✓
C13	32.98	C_17_H_17_O_3_N	284.1275	282.1116	2.29	261.0436; 164.0705; **147.0439**; 121.0648	*N*-*p-cis*-Coumaroyl tyramine	[40]	✓	✓	
C14	33.33	C_18_H_19_O_4_N	314.1382	312.1221	1.67	274.8365; 243.1029; 220.0979; 195.0847; **177.0551**; 145.0289; 121.0653	*N*-*cis*-feruloyl-tyramine	[8]	✓		✓
C15	33.47	C_17_H_17_O_3_N	284.1275	282.1116	2.29	261.0436; 164.0705; **147.0439**; 121.0648	*N*-*p-trans*-Coumaroyl tyramine *		✓	✓	
C16	33.62	C_28_H_31_O_8_N	510.2116	508.1944	1.36	462.1963; 325.1065; 210.0545; **177.0546**; 121.0648	canabisine-H	[36]	✓		
C17	33.81	C_18_H_19_O_4_N	314.1382	312.1221	1.51	244.0981; 220.0979; 194.0822; **177.0555**; 145.0292; 121.0653	*N*-*trans*-feruloyl-tyramine	[8]	✓	✓	✓
C18	34.02	C_19_H_19_O_5_N	344.1520	342.1323	1.25	282.3545; **177.0559**; 145.0295	A12 (feruloyl-tyramine derivatives)	[32]			✓
C19	34.98	C_36_H_37_O_9_N_2_	643.2638	641.2465	1.89	462.1903; 325.1063; 201.0544; **177.0545**; 121.0648	feruloyl-tyramine derivatives		✓		
C20	35.07	C_28_H_29_O_7_N	492.2010	490.1842	1.46	462.1909; **325.1066**; 293.0805; 201.0546; 175.0769; 121.0649	Lyciumamide C	[39]	✓		
C21	36.57	C_54_H_53_O_12_N_3_	936.3691		1.20	771.2914; 634.20612; **471.1429**; 375.0859; 263.0896; 203.5736; 121.0659	melongenamide D isomer	[41]	✓		
D1	24.74	C_33_H_40_O_2_1	773.2195	771.1937	1.21	611.140; 464.0762; **303.0325**; 163.0399;	Quercetin-3-*O*-Glu-7-*O*-Rha isomer	[42]		✓	
D2	26.89	C_27_H_30_O_17_	627.1610	625.1376	1.66	585.6912; **303.0520**; 285.0409; 257.043; 243.5646; 201.4349; 129.02379	Quercetin-3,7-*O*-diGlu	[42]	✓	✓	
D3	26.98	C_33_H_40_O_21_	773.2195	771.1940	1.84	726.3508; 559.7092; 465.1061; **303.0521**; 228.4964; 129.0548	Quercetin-3-*O*-Soph-7-*O*-Rha	[42]		✓	
D4	27.74	C_33_H_40_O_21_	773.2127	771.1940	1.08	611.3322; 472.2473; **303.0492**; 220.0965; 163.0399; 129.0541	Quercetin-3-*O*-Rut-7-*O*-Glu	[42]	✓		✓
D5	31.39	C_27_H_30_O_16_	611.1600	609.1417	1.03	449.1113; 465.1061; **303.0492**; 285.0413; 257.046; 201.0561129.05449	Rutin *		✓	✓	
D6	31.98	C_27_H_30_O_15_	595.1703	593.1472	1.63	**465.5521**; 329.0679; 287.0529; 258.2196; 243.5895; 230.3383; 129.0553	Kaempferol-3-*O*-Glu-7-*O*-Rha	[42]	✓	✓	
E1	33.67	C_45_H_72_O_17_	885.4835		0.87	867.4724; 415.3229; 299.2362; 271.2052; **253.1947**; 215.1792; 157.1011	Gracillin	[43]	✓		
E2	33.72	C_45_H_73_O_16_N	884.5084	882.4800	1.13	**866.4896**; 720.4325; 576.3898; 414.3403; 396.3259; 271.2054; 253.1949	Solasonine *		✓		
E3	33.77	C_51_H_84_O_22_	1049.5518		0.90	**887.4990**; 743.3851; 417.3355; 273.2207; 255.2107	parillin	[44]	✓		
E4	33.87	C_45_H_75_O_16_N	886.5152	884.4957	0.75	**868.5034**; 722.4475; 416.356; 398.3409; 273.2205; 255.2102; 173.1323	5,6-dihydrosolasonine *		✓		
E5	33.92	C_45_H_73_O_18_	903.5006		1.21	741.4469; 597.3309; 417.3389; 273.2230; **255.2124**; 145.0506	Timosaponin BIII	[45]	✓		
E6	34.49	C_47_H_77_O_17_N	928.5253		1.25	**458.3620**; 273.2207; 255.2102; 161.1323	Lycioside B	[1]	✓		
F1	3.93	C_9_H_11_O_2_N	166.0867		1.10	153.0416; 142.9681; **120.0814**; 103.0548	Phenylalanine isomer	[1]	✓	✓	✓
F2	5.51	C_9_H_11_O_3_N	166.0877		1.13	142.9681; 138.0554; **120.0812**; 103.0548	Phenylalanine isomer		✓	✓	✓
F3	9.95	C_11_H_9_O_2_N	188.0721		1.95	170.0613; **146.0412**; 118.0611	3-amino-2-naphthoic acid	[46]	✓	✓	✓
F4	10.44			371.0991	0.11	205.0506; **163.0398**; 145.9288; 119.0498			✓		
F5	31.16			568.3110		539.2707; 363.2378; **268.0598**; 135.0436					✓
F6	31.29		748.3361			609.3981; 399.1691; 360.1675; **314.1518**; 215.0829; 171.0929; 136.0770					✓
F7	31.69			630.3469		**498.3042**; 469.2647; 387.2245; 241.0723; 151.2698					✓
F8	32.56			298.1066		256.0959; 178.0492; **135.0435**					✓
F9	32.91			647.3257		618.1393; 483.2572; **412.2080**; 395.2055; 161.4154					✓
F10	34.22		897.3956	895.3695		879.3847; 689.3096; 486.2017; **468.1911**; 422.583; 395.1742; 159.0929					✓
F11	34.23			898.4049		690.3203; **527.2580**; 387.1645; 203.0810; 153.0655					✓

^1^ RT represents retention time (min); ^2^ MS/MS fragments only show the fragmentation ions in positive ionization mode except that the [M + H]^+^ ion of the compound was not detected (show the fragmentation ions in negative ionization mode); the number in **bold** means the most abundant product ion. ^3^ Ref. represents the references; ^4–6^ F, L, R represent fruits, leaves and Root barks respectively. * Represents the compounds confirmed by standards.

**Table 2:** The amounts of seven standards in 1 g dry fruits, leaves and root barks.

Compounds	Fruits (μg/g)	Leaves (μg/g)	Root Barks (μg/g)
Kukoamine B	-	-	10,900 ± 3
Scopolin	12.7 ± 0.08	-	-
Chlorogenic acid	-	1577 ± 4	-
Rutin	93 ± 5	663 ± 15	-
Solasonine	2.16 ± 0.02	-	-
5,6-Dihydrosolasonine	43 ± 3	-	-
*N*-*p-trans*-Coumaroyltyramine	3.33 ± 0.02	14 ± 1	-

Results are expressed as means ± SD, *n* = 3.

**Table 3:** Antioxidant capacity of fruit, leaf and root bark extracts.

Extracts	DPPH(IC_50_) ^1^ (µg/mL)	ABTS(IC_50_) ^2^ (µg/mL)	FRAP(RC_50_) ^3^ (µg/mL)
Fruits	1974.3 ± 0.4	247.0 ± 0.8	725 ± 1.4
Leaves	123.5 ± 0.5	56 ± 1	192.6 ± 0.02
Root barks	85.0 ± 0.3	40.7 ± 0.4	224 ± 1
Ascorbic acid ^4^	11.2 ± 0.5	-	-
Trolox ^5^	-	6 ± 1	37.7 ± 0.3

^1^ DPPH (IC_50_) represents the extract concentration scavenging 50% of DPPH radical; ^2^ ABTS (IC_50_) represents the extract concentration scavenging 50% of ABTS radical; ^3^ FRAP (RC_50_) represents the extract concentration providing 50% reduction of Fe^3+^ to Fe^2+^; ^4,5^ represent the positive control; Results are expressed as means ± SD, *n* = 3.

## Supplementary Materials

The following are available online, Figure S1: Total ion chromatograms (TICs) of 7 standard substances in the positive ion mode; Figure S2: Proposed fragmentation pathway for solasonine, based on HR-Orbitrap MS/MS spectra; Figure S3. The HR-Orbitrap MS/MS spectra and Proposed fragmentation pathway of chlorogenic acid; Figure S4. The HR-Orbitrap MS/MS spectra and proposed fragmentation pathway of scopolin; Figure S5. The HR-Orbitrap MS/MS spectrum and the proposed fragmentation pathway of kukoamine B; Figure S6. The HR-Orbitrap MS/MS spectrum and the proposed fragmentation pathway of *N*-*p-trans*-Coumaroyl tyramine; Figure S7. The HR-Orbitrap MS/MS spectrum and the proposed fragmentation pathway of rutin; Figure S8. HR-Orbitrap MS/MS spectrum of A1; Figure S9. HR-Orbitrap MS/MS spectrum of B52; Figure S10. HR-Orbitrap MS/MS spectra of B63; Figure S11. HR-Orbitrap MS/MS spectrum of B18; Figure S12. HR-Orbitrap MS/MS spectra of compounds B46, B50 and B54; Figure S13. HR-Orbitrap MS/MS spectra of B51; Figure S14. HR-Orbitrap MS/MS spectra of C15; Figure S15. HR-Orbitrap MS/MS spectrum of D6; Figure S16. HR-Orbitrap MS/MS spectrum of E3; Figure S17. Protection Effect of the extracts from fruits (0.25 mg/mL), leaves and root barks (0.2 mg/mL) on 100 µM H_2_O_2_-induced intracellular ROS production in L02 cells. Table S1.13C NMR spectral data for aglycone and glycoside moieties of solasonine (SS) and 5.6-dihydrosolasonine (2HSS) in pyridine (D5); Table S2. The regression equation, LOD, LOQ, intra-day and inter-day of the 7 standards using the optimized method for calibration; Table S3. The recoveries of 7 standards (n = 3); Table S4. MS/MS parameters for the construction of the database of the 91 compounds in fruit of *L. barbarum* using UPLC- Qtrap-MS in the positive ion mode.
