# Supplementary material for: Comparative Study of the Chemical Constituents and Bioactivities of the Extracts from Fruits, Leaves and Root Barks of Lycium barbarum
Source: Molecules. 2019 Apr 22;24(8):1585. doi: 10.3390/molecules24081585 (PMC6514792; doi:10.3390/molecules24081585)
Supplement: Supplementary file 1 [file molecules-24-01585-s001.pdf]

Supplementary Materials: Comparative study of the chemical constituents and bioactivities of the extracts from fruits, leaves and root barks of *Lycium barbarum*

Xiao Xiao[1,2,†], Wei Ren[1,2,†], Nan Zhang[1,2], Tao Bing[1,2], Xiangjun Liu[1,2], Zhenwen Zhao[1,2,*], Dihua Shangguan[1,2,*]

[1] Beijing National Laboratory for Molecular Sciences, Key Laboratory of Analytical Chemistry for Living Biosystems, CAS Research/Education Center for Excellence in Molecular Sciences, Institute of Chemistry, Chinese Academy of Sciences, Beijing, 100190, China

[2] University of the Chinese Academy of Sciences, Beijing 100049, China

[†] These authors contributed equally to this work.

[*] To whom correspondence should be addressed: *e-mail: zhenwenzhao@iccas.ac.cn, sgdh@iccas.ac.cn

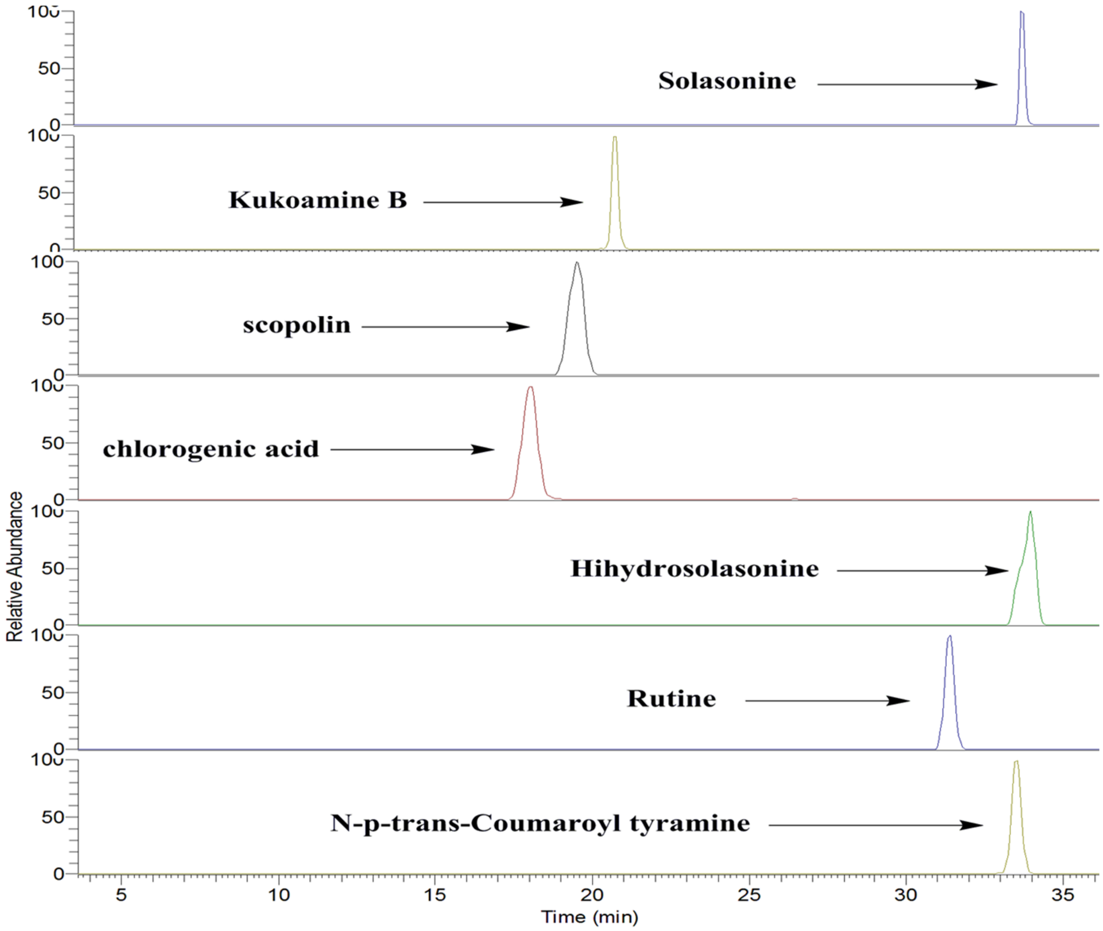

Figure S1. Total ion chromatograms (TICs) of 7 standard substances in the positive ion mode.

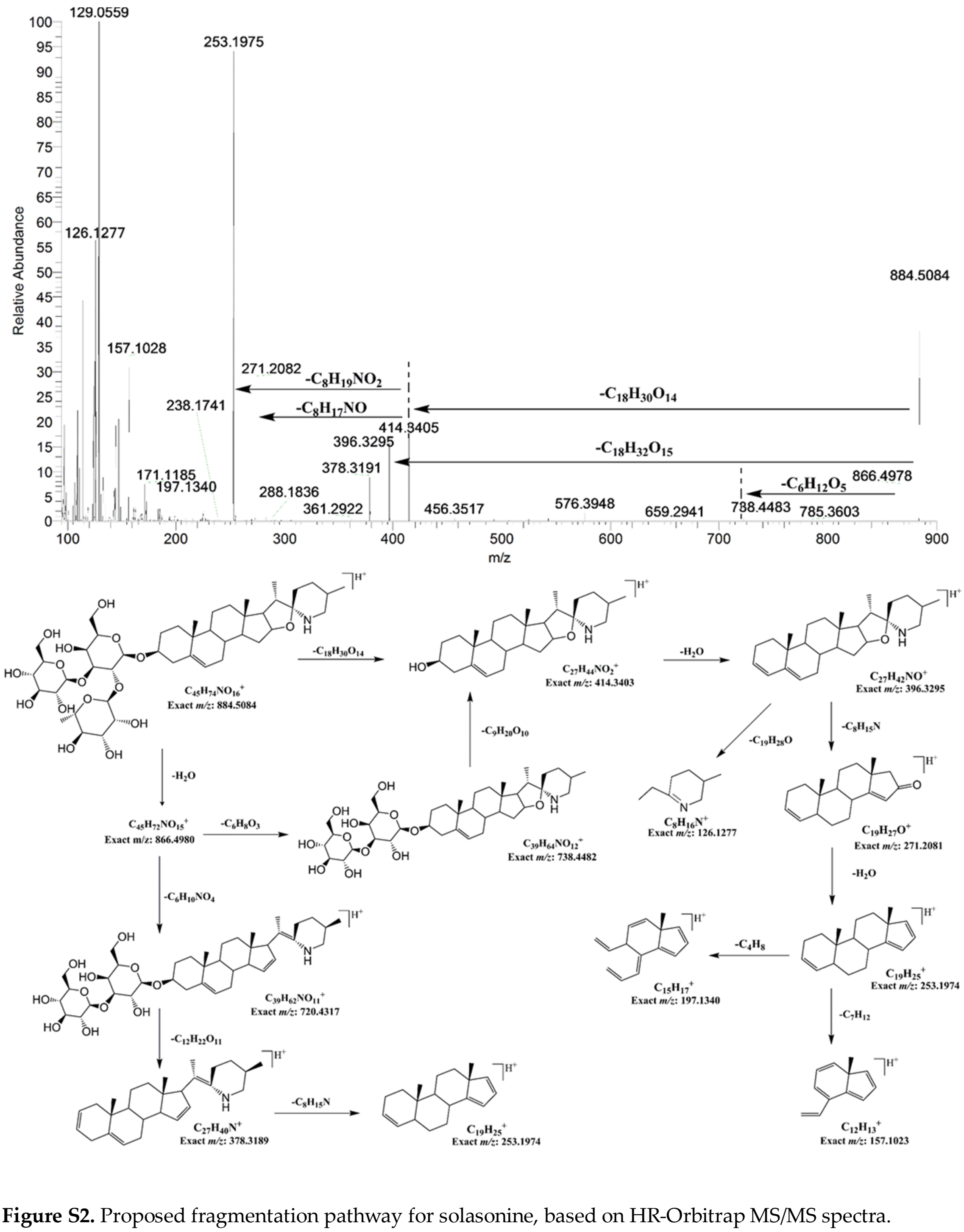

Figure S2. Proposed fragmentation pathway for solasonine, based on HR-Orbitrap MS/MS spectra.

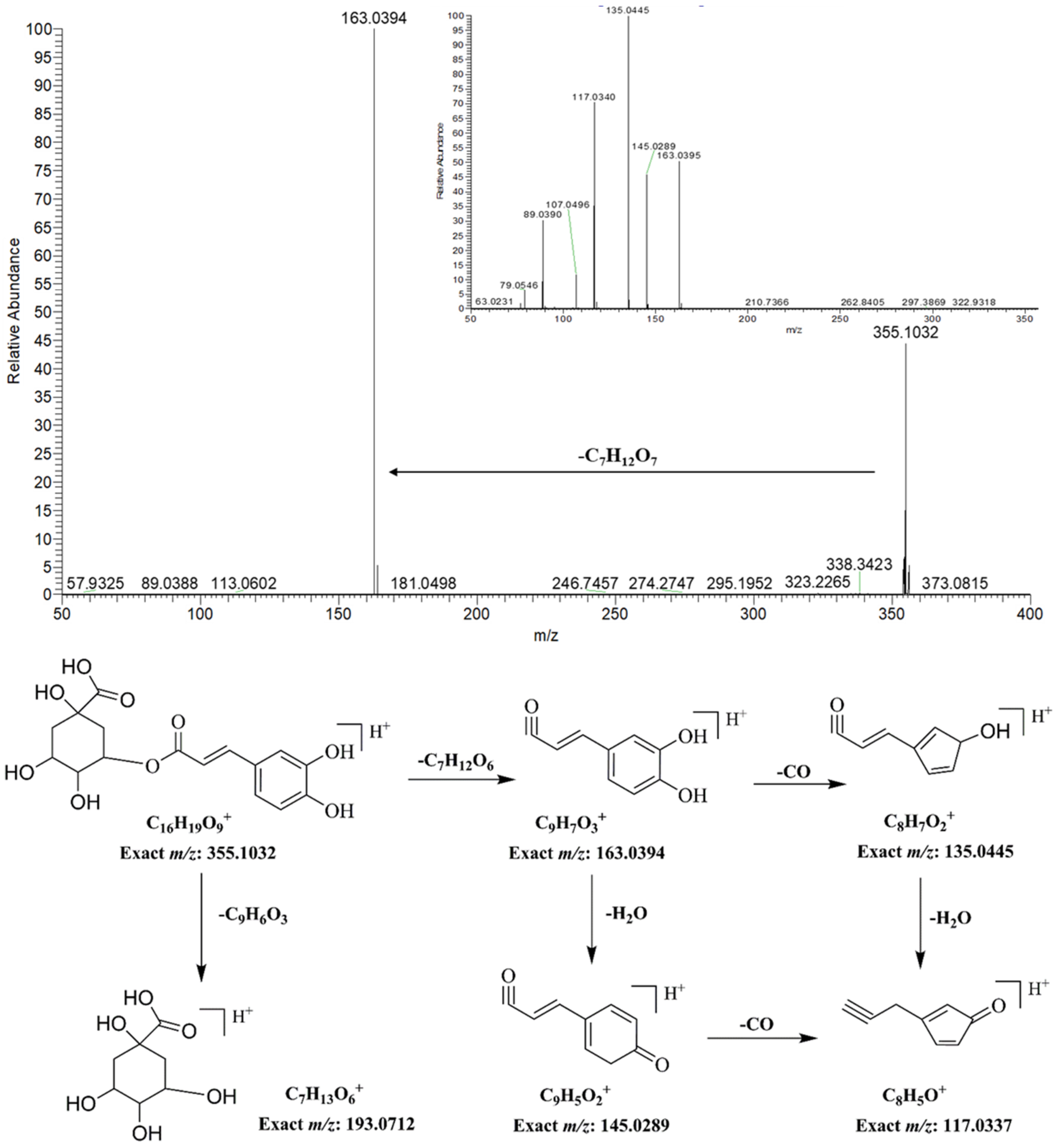

Figure S3. The HR-Orbitrap MS/MS spectra and Proposed fragmentation pathway of chlorogenic acid.

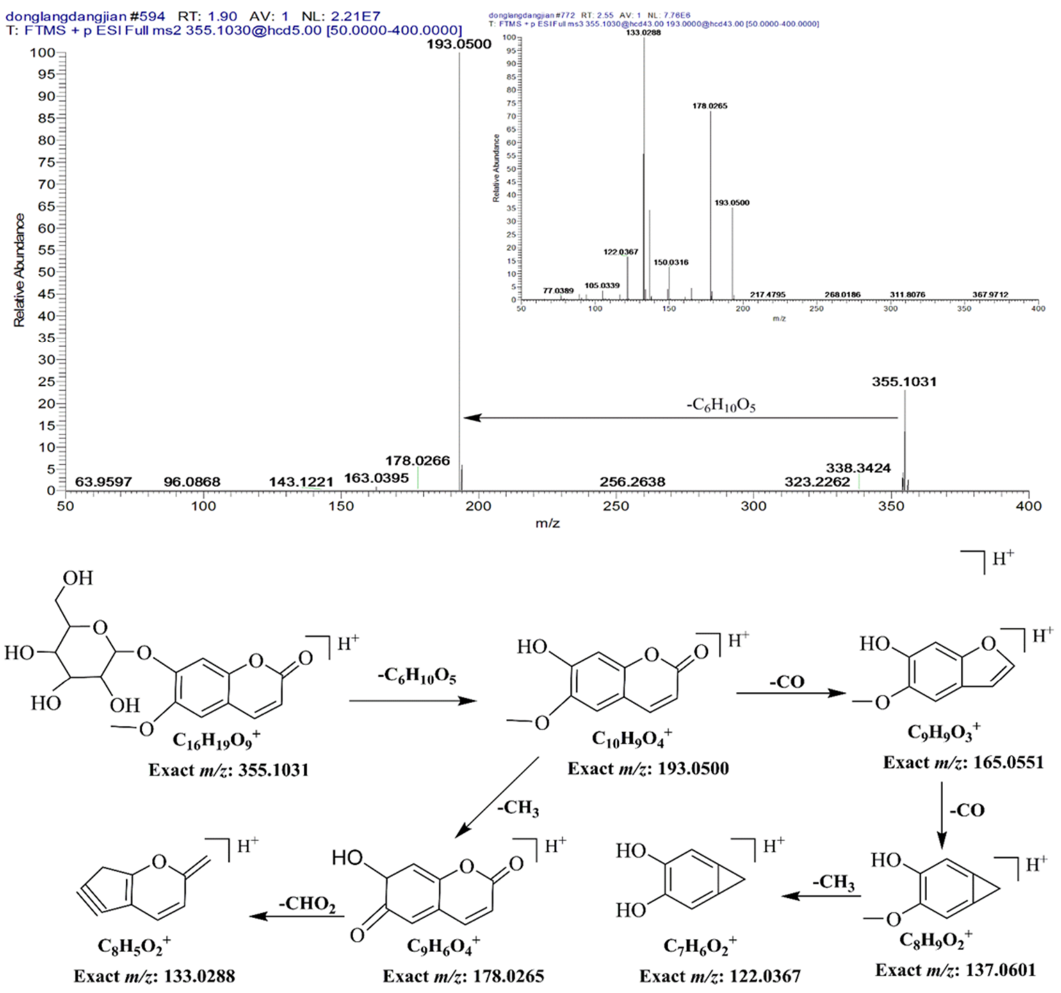

Figure S4. The HR-Orbitrap MS/MS spectra and proposed fragmentation pathway of scopolin.

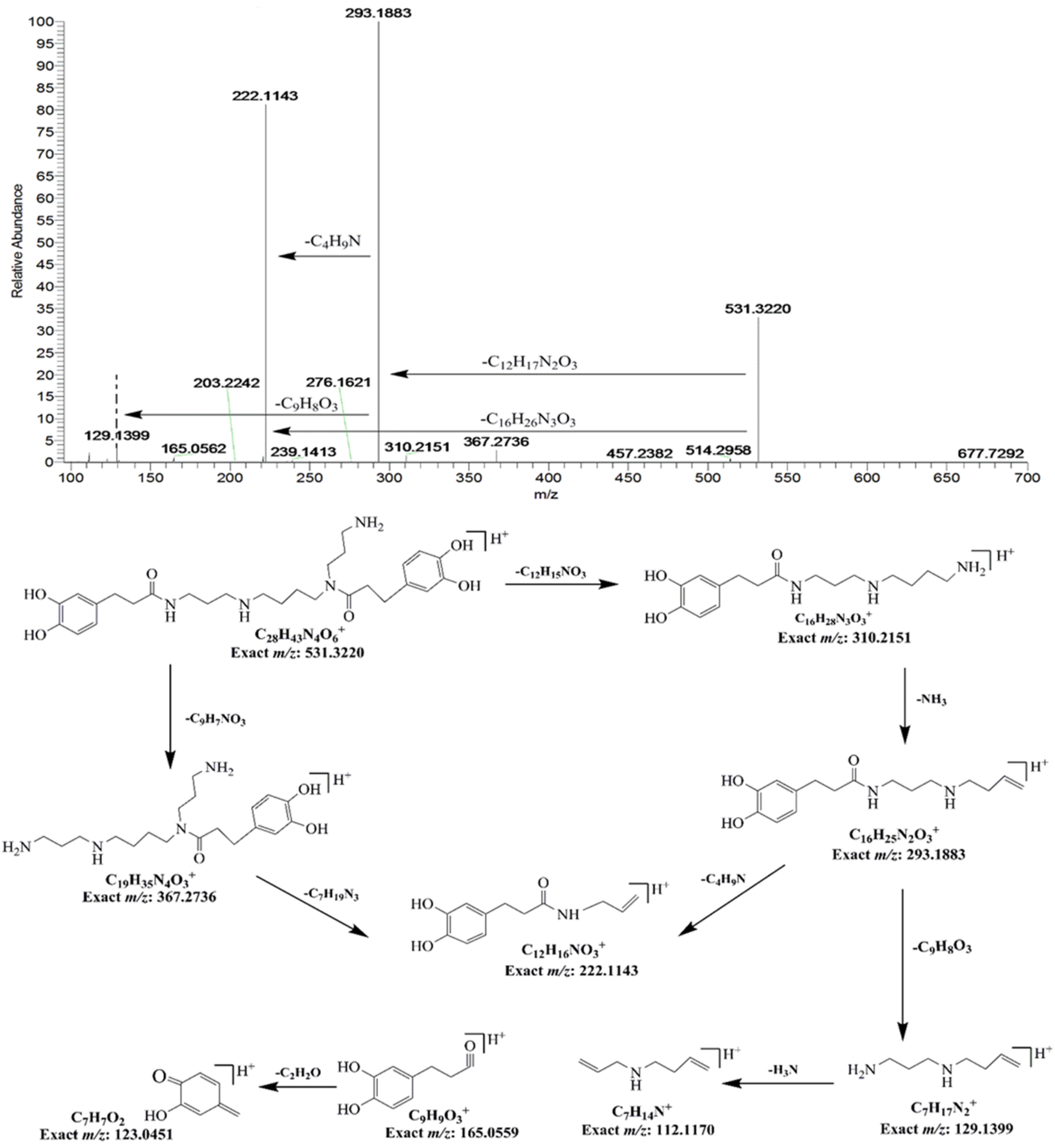

Figure S5. The HR-Orbitrap MS/MS spectrum and the proposed fragmentation pathway of kukoamine B.

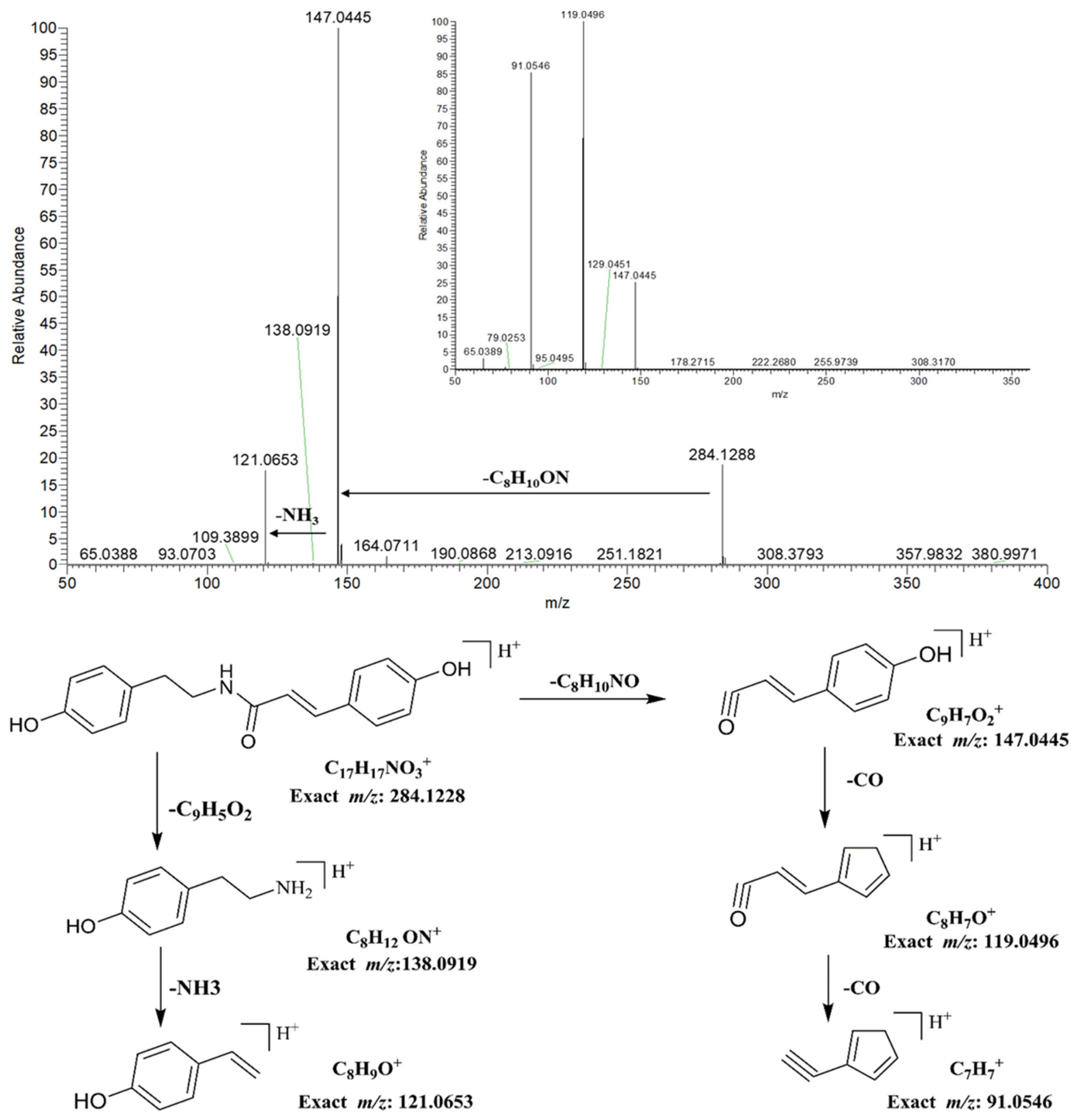

Figure S6. The HR-Orbitrap MS/MS spectrum and the proposed fragmentation pathway of N-p-trans-Coumaroyl tyramine.

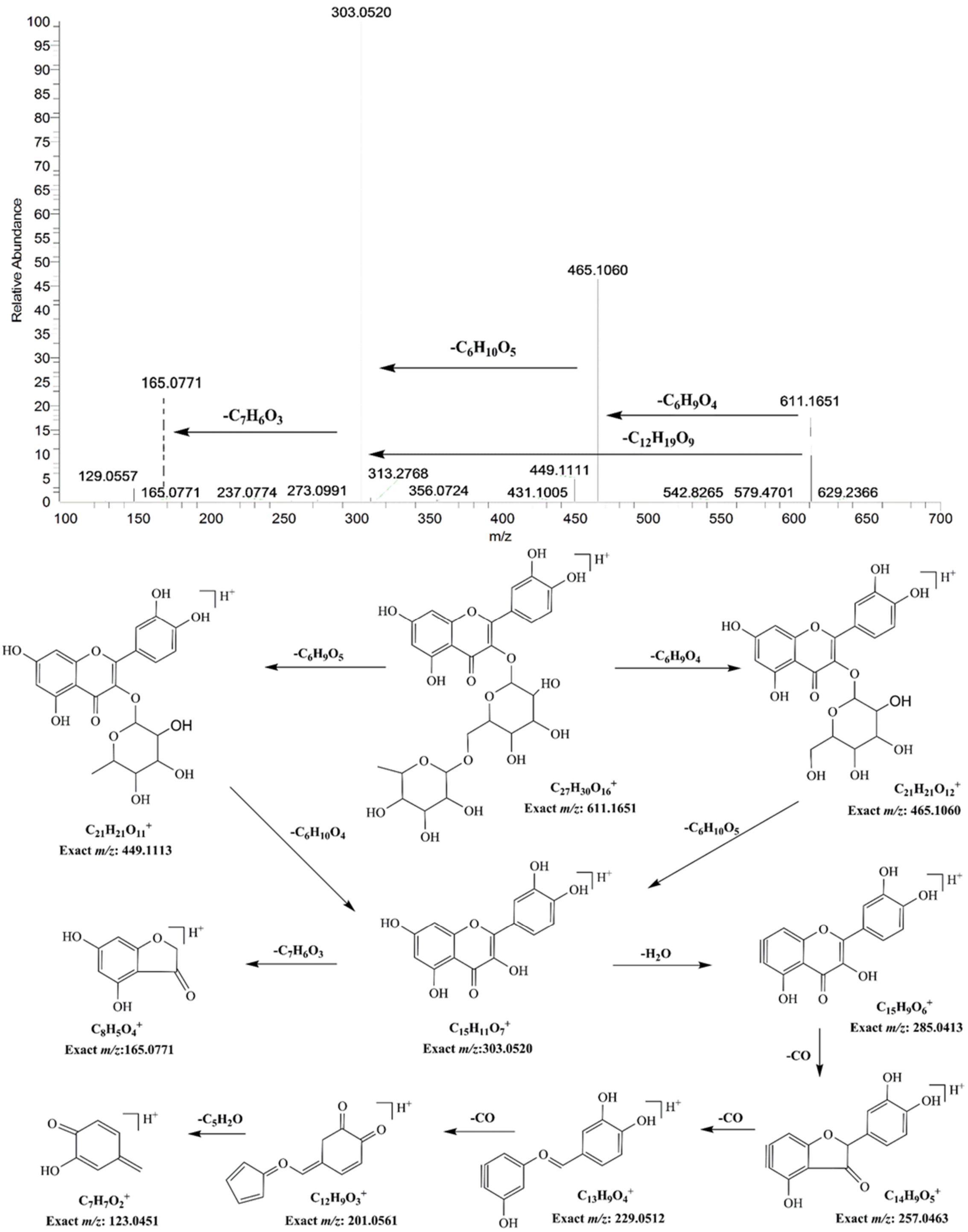

Figure S7. The HR-Orbitrap MS/MS spectrum and the proposed fragmentation pathway of rutin.

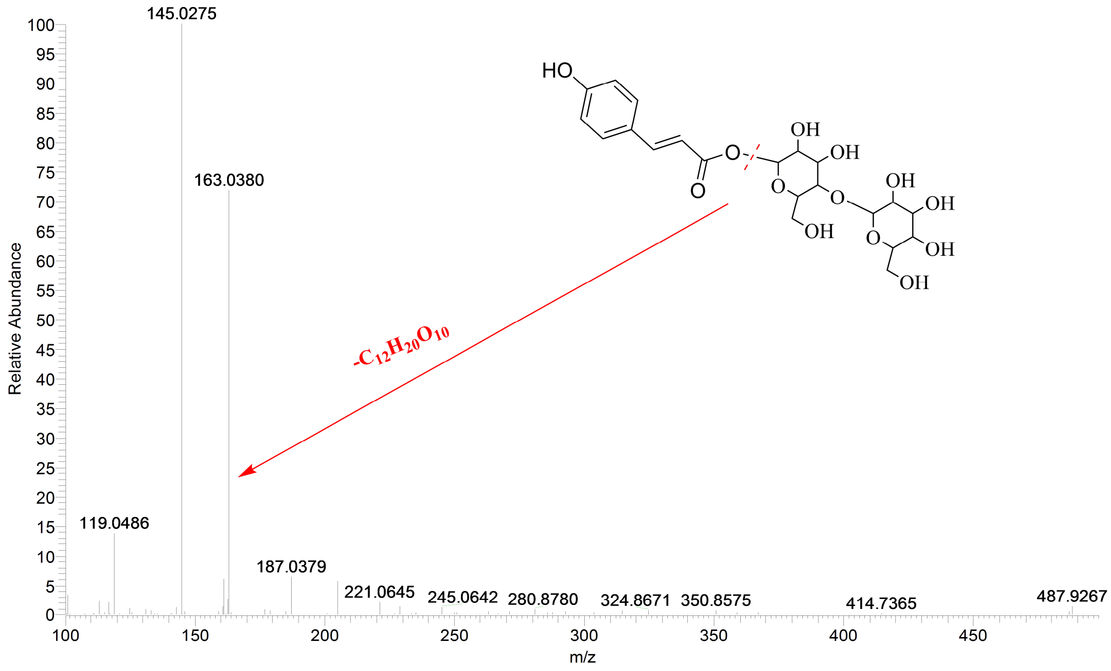

Figure S8. HR-Orbitrap MS/MS spectrum of A1.

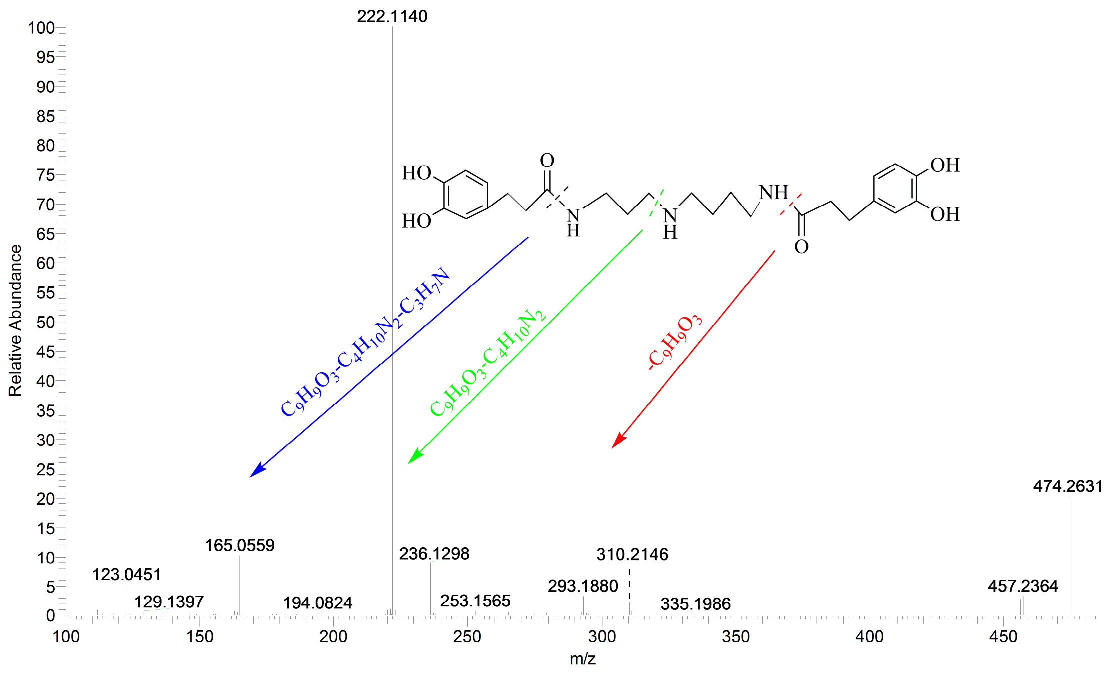

Figure S9. HR-Orbitrap MS/MS spectrum of B52.

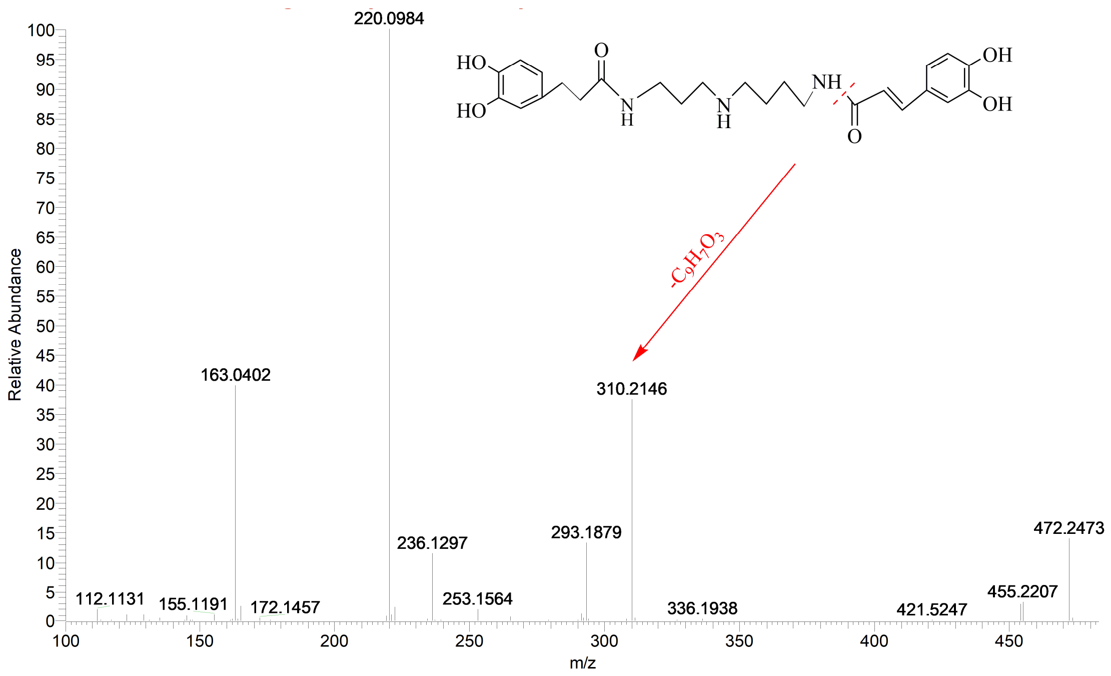

Figure S10. HR-Orbitrap MS/MS spectra of B63.

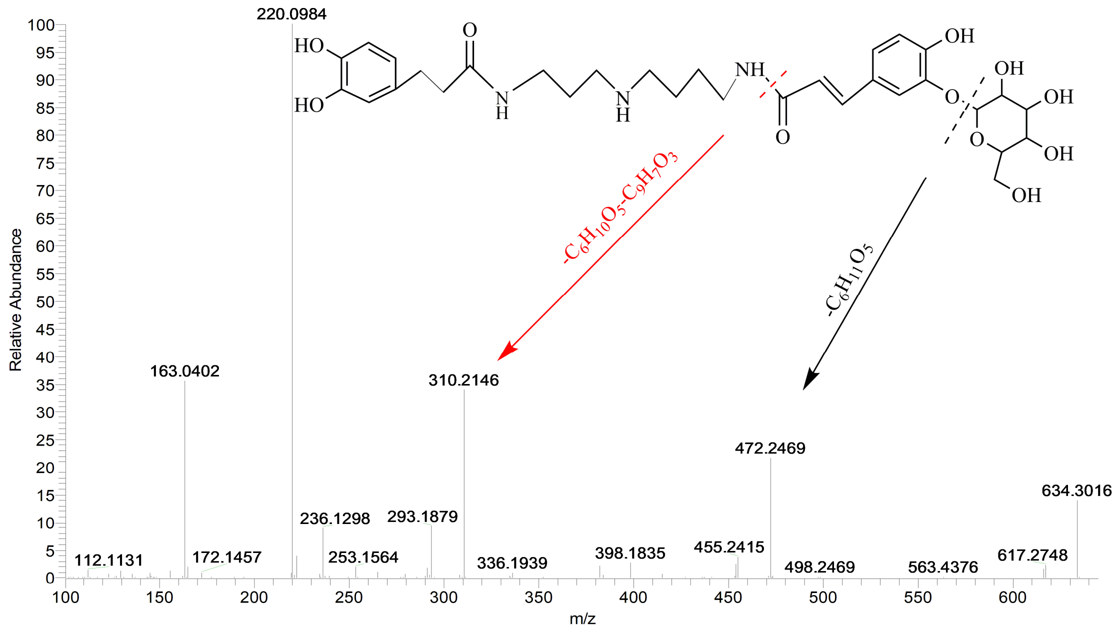

Figure S11. HR-Orbitrap MS/MS spectrum of B18.

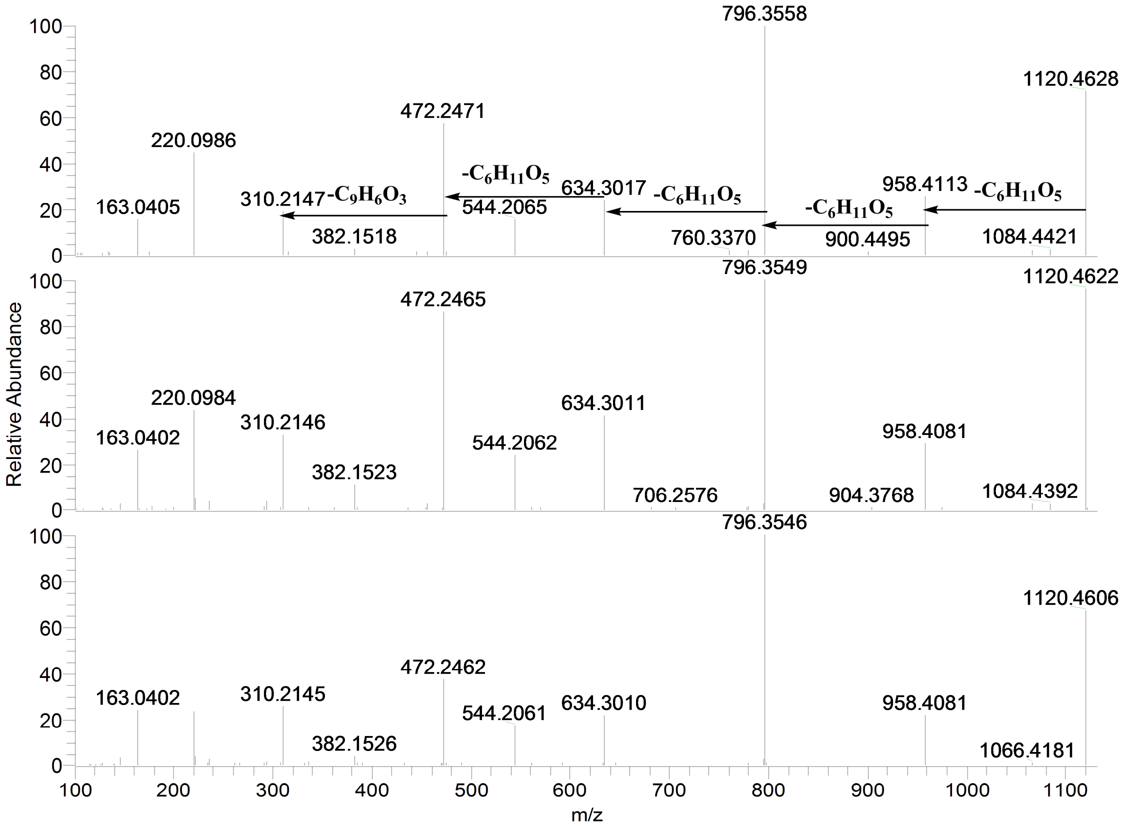

Figure S12. HR-Orbitrap MS/MS spectra of compounds B46, B50 and B54.

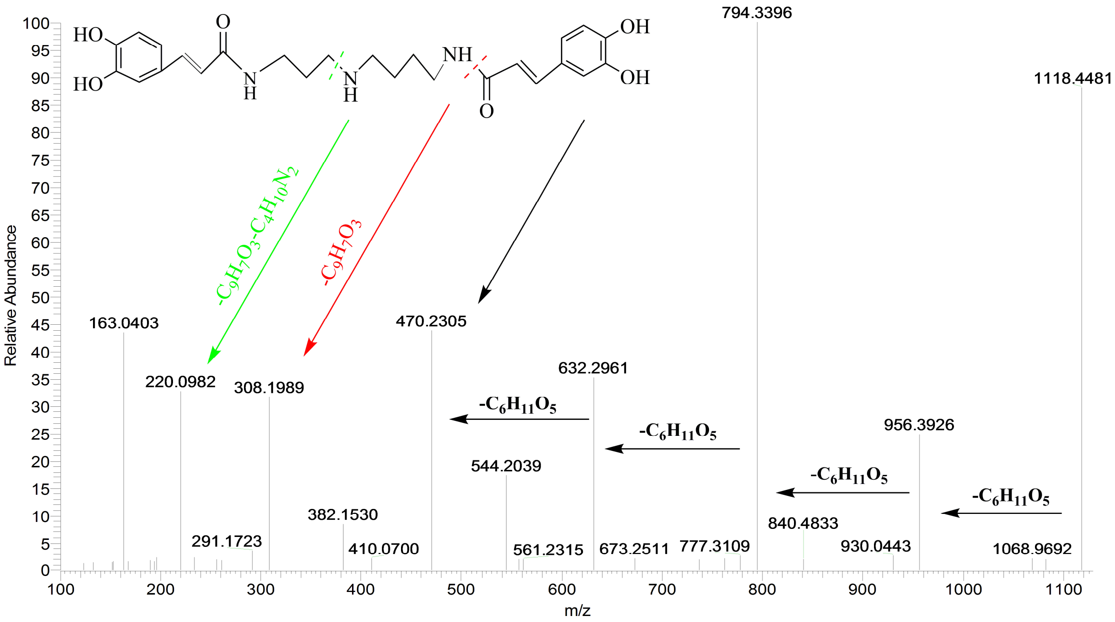

Figure S13. HR-Orbitrap MS/MS spectra of B51.

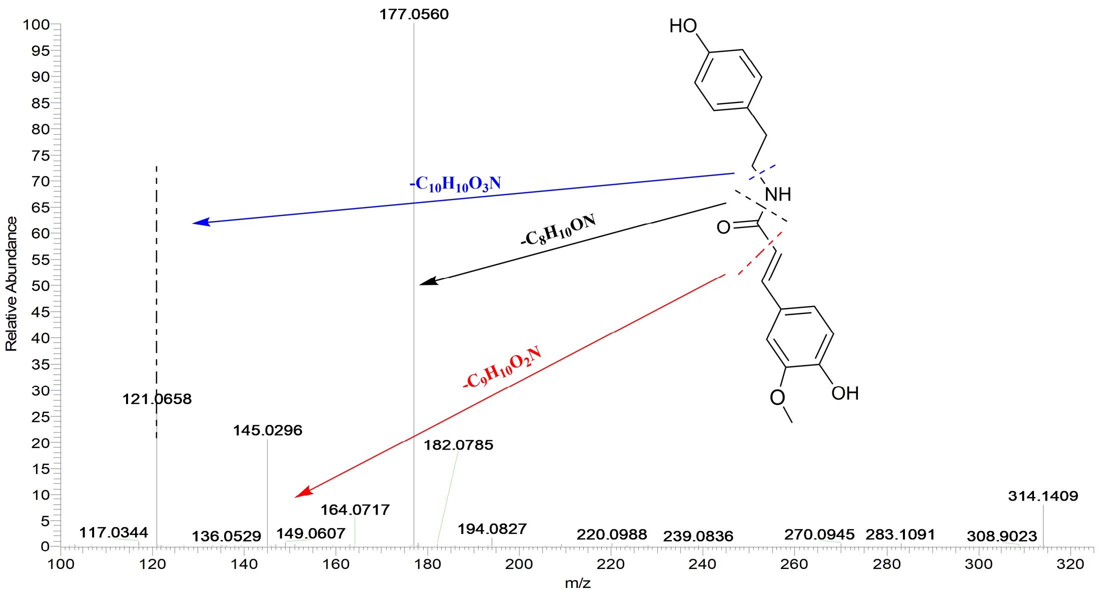

Figure S14. HR-Orbitrap MS/MS spectra of C15.

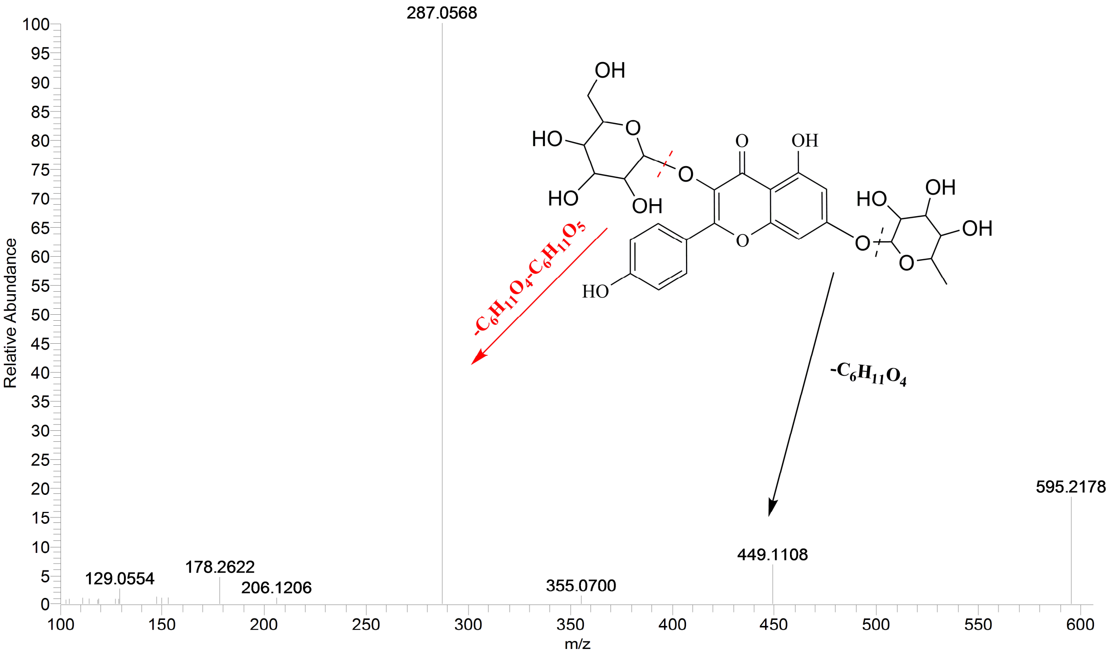

Figure S15. HR-Orbitrap MS/MS spectrum of D6.

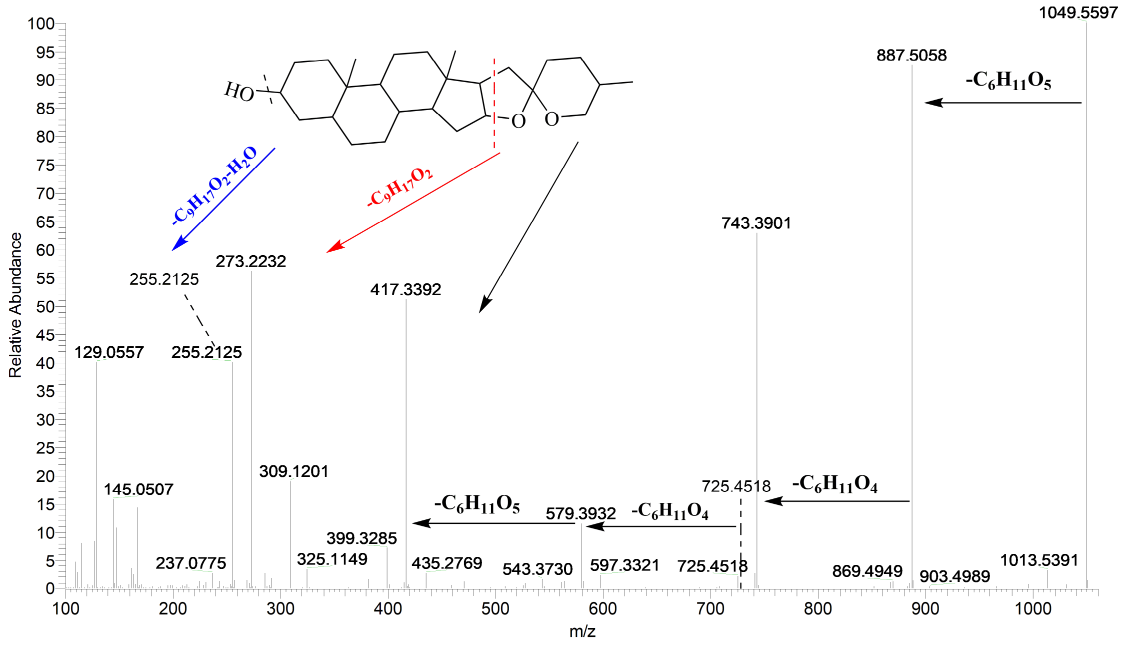

Figure S16. HR-Orbitrap MS/MS spectrum of E3.

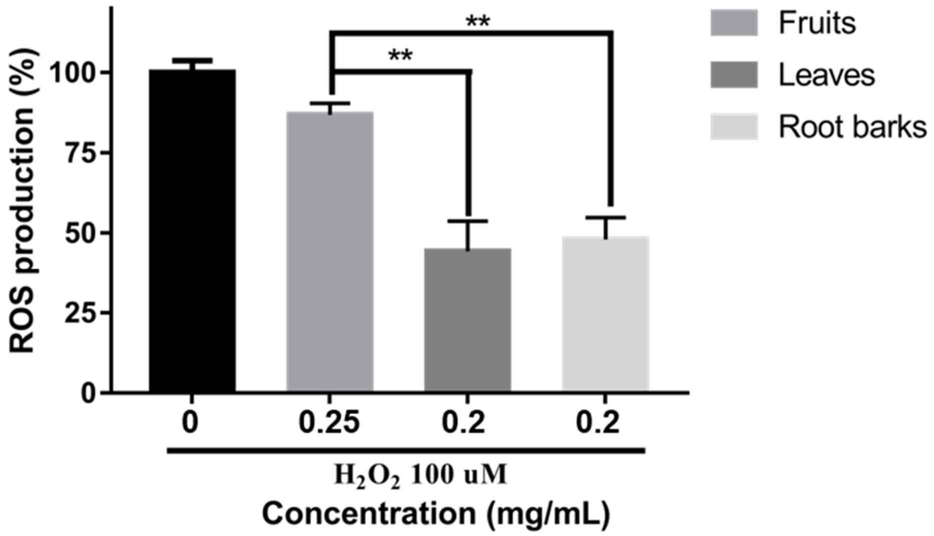

Figure S17. Protection Effect of the extracts from fruits (0.25 mg/mL), leaves and root barks (0.2 mg/mL) on 100 μM H_2O_2-induced intracellular ROS production in L02 cells. Results were expressed as means ± SD, n=3. **p < 0.01.

Table S1. 13C NMR spectral data for aglycone and glycoside moieties of solasonine (SS) and 5.6-dihydrosolasonine (2HSS) in pyridine (C5D5N).

C	SS	2HSS	C	SS	2HSS
1	37.20	37.20	24	31.84	30.86
2	29.89	30.19	25	31.43	31.17
3	78.13	78.47	26	45.68	45.87
4	38.94	33.73	27	19.14	19.92
5	140.60	43.06	gal-1	100.17	100.81
6	121.40	28.77	2	76.26	76.26
7	32.26	33.38	3	84.59	83.40
8	31.84	31.82	4	70.22	70.70
9	49.76	48.85	5	77.45	79.58
10	36.85	35.69	6	62.30	63.39
11	20.71	20.77	glu-1	105.63	106.50
12	38.54	38.49	2	62.34	63.39
13	40.75	41.45	3	78.13	78.47
14	56.04	55.67	4	71.36	70.70
15	32.82	32.20	5	78.13	79.76
16	78.27	79.02	6	62.34	63.39
17	62.20	63.31	rha-1	101.98	103.20
18	18.38	17.96	2	72.62	64.67
19	19.14	13.70	3	74.72	73.68
20	41.97	42.30	4	74.90	75.38
21	15.87	15.53	5	69.20	72.53
22	98.41	99.64	6	14.85	13.70
23	32.82	33.73			

Table S2. The regression equation, LOD, LOQ, intra-day and inter-day of the 7 standards using the optimized method for calibration.

Compounds	Regression equation	R^2	Linearity range (μg/mL)	LOD (μg/mL)	LOQ (μg/mL)	intra-day（n=3）		Inter-day(n = 6)	
						Concentration (μg/mL)	RSD (%)	Concentration (μg/mL)	RSD (%)
KukoamineB	Y =235181.48X-623985.10	0.998	2.69-100.88	0.08	0.26	4.9±0.01	0.2	4.9±0.02	0.4
Rutin	Y =44449.05X-3341.72	0.995	0.36-49.23	0.02	0.07	43.8±0.8	1.7	43.9±0.6	1.4
Solasonine	Y =6888.68X-848.10	0.989	0.72-25.31	0.04	0.12	22.3±0.6	2.9	22.4±0.5	2.4
Dihydrosolasonine	Y =3972.51X-12740.89	0.993	3.59-99.03	0.10	0.32	20.2±0.3	1.7	22.9±0.3	1.4
Chlorogenic acid	Y = 165955.13X-390952.12	0.989	31.94-207.23	0.07	0.23	23.2±1.9	4.6	23.4±1.7	3.9
Scopolin	Y = 265223.92X-162882.35	0.994	0.72-25.20	0.02	0.06	33.8±0.2	1.3	34.4±0.9	3.5
N-p-trans-coumaroyltyramine	Y = 267005.14X-107456.73	0.990	0.22-25.01	0.06	0.2	2.3±0.4	1.5	2.8±0.6	2.8

Table S3. The recoveries of 7 standards (n = 3)

compounds	Initial amount (ug/mL)	Added amount (ug/mL)	Total recovered amount (ug/mL)	Recoveryb (%)	RSD (%)
kukoamineB	0	1.25	1.25±0.01	99.79	0.44
	0	2.50	2.46±0.02	98.57	0.86
	0	5.00	5.01±0.04	100.24	0.72
rutin	4.65	1.25	6.27±0.17	101.85	2.80
	4.65	2.50	7.49±0.06	99.79	0.84
	4.65	5.00	10.17±0.15	103.29	1.45
5,6-dihydrosolasonine	2.15	1.25	3.19±0.05	107.69	1.42
	2.15	2.50	4.43±0.24	103.55	5.33
	2.15	5.00	6.26±0.06	99.07	0.65
solasonine	0.11	1.25	2.07±0.01	100.67	2.80
	0.11	2.50	3.13±0.13	92.69	0.84
	0.11	5.00	6.01±0.24	103.98	7.01
scopolin	0.64	1.25	2.07±0.06	100.36	3.55
	0.64	2.50	3.28±0.07	100.22	2.10
	0.64	5.00	5.65±0.03	99.52	0.58
chlorogenic acid	0	1.25	1.26±0.01	98.88	0.40
	0	2.50	2.64±0.06	97.67	2.28
	0	5.00	5.14±0.08	96.21	1.52
N-p-trans-coumaroyltyramine	0.17	1.25	2.20±0.01	112.25	0.40
	0.17	2.50	3.26±0.04	98.34	1.09
	0.17	5.00	5.71±0.10	98.12	1.70

Table S4. MS/MS parameters for the construction of the database of the 91 compounds in fruit of *L. barbarum* using UPLC- Qtrap-MS in the positive ion mode.

No.	R	Q1	Q3	Declustering potential (V)	Collision energy (eV)	No.	R	Q1	Q3	Declustering potential (V)	Collision energy (eV)
A5	15.45	355.1	163	180	43	B52	29.40	474.3	222	180	40
A7	16.88	355.1	163	180	43	B53	29.44	912.4	163	200	89
A10	18.87	355.1	193	140	45	B54	29.58	1120.5	796	200	58
A12	20.42	355.1	163	180	43	B55	29.59	634.3	220	200	51
B2	18.48	855.4	455	200	61	B56	29.60	632.3	220	200	51
B3	19.3	855.4	455	200	61	B57	29.64	912.4	163	200	89
B4	19.39	1017.5	617	200	47	B58	29.71	796.3	472	200	57
B5	19.50	531.3	293	180	39	B59	29.71	794.3	470	200	60
B6	19.59	693.4	293	200	60	B60	29.74	750.3	310	180	61
B8	20.60	1017.5	617	200	47	B61	29.74	1118.4	632	200	58
B10	21.02	531.3	293	180	39	B62	29.88	750.3	310	180	61
B12	23.31	529.3	291	180	36	B63	30.03	472.2	163	180	54
B13	23.83	958.4	472	200	60	B64	30.13	634.3	220	200	51
B14	24.41	794.3	470	200	60	B67	30.14	794.3	470	200	60
B15	24.41	960.4	474	200	59	B70	30.56	470.2	220	180	46
B16	24.75	798.4	474	200	57	B73	30.71	963.4	384	200	52
B17	24.78	796.3	472	200	57	C1	8.30	251.1	163	200	32
B18	24.80	634.3	220	200	51	C2	10.26	251.1	163	200	32
B19	24.94	958.4	472	200	60	C5	30.22	476.2	177	180	50
B20	25.17	798.4	474	200	57	C6	30.42	648.3	234	200	47

No.	R	Q1	Q3	Declustering potential (V)	Collision energy (eV)	No.	R	Q1	Q3	Declustering potential (V)	Collision energy (eV)
B24	25.77	958.4	472	200	60	C8	31.34	486.3	234	180	40
B25	26.02	798.4	474	200	57	C9	31.83	476.2	122	180	50
B26	26.10	796.3	472	200	57	C10	32.09	625.3	325	200	34
B27	26.36	960.4	474	200	59	C11	32.22	498.3	184	180	34
B28	26.41	958.4	472	200	60	C13	32.98	284.1	146	180	34
B29	26.44	798.4	474	200	57	C14	33.33	314.1	145	180	33
B30	26.79	958.4	472	200	60	C15	33.47	284.1	146	180	34
B31	27.02	634.3	220	200	51	C16	33.62	510.2	177	180	51
B32	27.03	632.3	220	200	51	C17	33.81	314.1	145	180	33
B33	27.23	958.4	472	200	60	C19	34.98	643.3	177	180	61
B34	27.42	960.4	474	200	59	C20	35.07	492.2	325	180	31
B35	27.51	636.3	474	200	51	C21	36.57	936.4	634	200	69
B36	27.73	632.3	220	200	51	D3	27.23	773.2	303	200	66
B37	27.85	794.3	470	200	60	D4	31.07	773.2	303	200	66
B38	27.91	958.4	472	200	60	D5	31.39	611.2	303	180	48
B39	28.04	796.3	472	200	57	D6	31.98	595.2	287	115	29
B40	28.16	956.4	470	200	68	E1	33.67	885.5	271	200	61
B41	28.30	960.4	474	200	59	E2	33.72	884.5	396	200	51
B42	28.57	634.3	220	200	51	E3	33.77	1049.6	273	200	49
B44	28.77	912.4	163	200	89	E4	33.87	886.5	398	200	52
B46	29.06	1120.5	796	200	58	E5	33.92	903.5	273	200	47
B47	29.11	912.4	163	200	89	E6	34.49	928.5	458	200	27

No.	R	Q1	Q3	Declustering potential (V)	Collision energy (eV)	No.	R	Q1	Q3	Declustering potential (V)	Collision energy (eV)
B48	29.16	634.3	220	200	51	F1	3.93	166.1	120	200	35
B49	29.23	632.3	220	200	51	F2	5.51	166.1	120	200	35
B50	29.29	1120.5	796	200	58	F3	9.95	188.1	146	180	30
B51	29.32	794.3	470	200	60						
